# Biotransformation and partitioning of structurally different PFAS by wastewater microbial consortia

**DOI:** 10.1039/d5ew00528k

**Published:** 2025-10-27

**Authors:** Sumaiya Saifur, Nisa Vyverberg, John Michael Aguilar, Jonathan Antle, Nirupam Aich, Diana S. Aga, Ian M. Bradley

**Affiliations:** a Department of Civil, Structural and Environmental Engineering, University at Buffalo – The State University of New York USA ianbradl@buffalo.edu +1 (716) 645 4004; b Department of Chemistry, University at Buffalo – The State University of New York USA; c Department of Civil and Environmental Engineering, University of Nebraska-Lincoln USA; d The Research and Education in Energy, Environment, and Water (RENEW) Institute, University at Buffalo – The State University of New York USA

## Abstract

Water resource recovery facilities (WRRFs) are sinks of legacy and replacement per- and polyfluoroalkyl substances (PFAS). This study evaluates the potential biotransformation, bioaccumulation, and adsorption of PFAS in wastewater sludge. Individual partitioning of parent PFAS and transformation products were measured in aqueous and solid phases of aerobic and anaerobic bacterial cultures for five structurally variable legacy and replacement PFAS using independent tests: perfluorooctanoic acid (PFOA), perfluorooctane sulfonic acid (PFOS), perfluorobutane sulfonic acid (PFBS), 6:2 fluorotelomer sulfonate (6:2 FTS), and hexafluoropropylene oxide dimer acid (GenX). Anaerobic cultures (anaerobic digestate and dehalogenating KB-1®) showed only adsorption (10.9–38.3%) with no transformation of the parent PFAS, irrespective of structural variances, in 90 days. Aerobic cultures from activated and nitrification sludge resulted in adsorption (26.9 ± 1.2–55.8 ± 1.4%), biotic accumulation (13.35–17.55%), and transformation (28.96–47.87%) of long-chain PFAS in 21 days. Notably, PFOA, PFOS, and 6:2 FTS were rapidly transformed 47.87 ± 1.6%, 28.96 ± 0.6%, and 43.1 ± 1.0%, respectively, after a shift occurred in microbial community structure under batch growth after 6 days, with the generation of shorter-chain compounds (carboxylates and sulfonates) and limited defluorination. Aerobic wastewater microbial communities converged, with *Methylophilus*, *Acidomonas*, *Pseudomonas*, *Clostridium*, *Klebsiella*, and *Acinetobacter* positively correlated with PFAS degradation. This study highlights the importance of unit processes and microbial community structure in controlling the fate and transport of select PFAS.

## Abstract 2

Water resource recovery facilities (WRRFs) are sinks of legacy and replacement per- and polyfluoroalkyl substances (PFAS).

## Graphical abstract

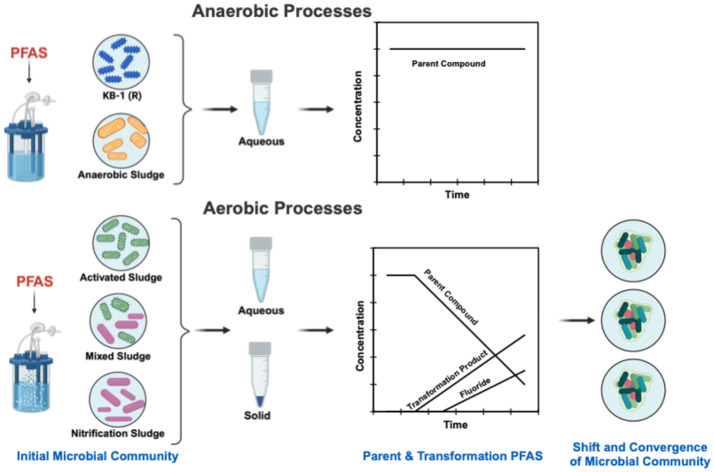

**Water impact**

The partitioning, fate, and transport of PFAS in wastewater effluent and biosolids may be significantly different across different types of wastewater treatment. Here, anaerobic and aerobic wastewater processes were investigated. While anaerobic cultures suggested no significant bioaccumulation or transformation of any parent PFAS tested, aerobic cultures from traditional wastewater treatment resulted in significant transformation and chain-shortening of long-chain legacy PFAS.

## 1. Introduction

Per- and polyfluoroalkyl substances, or PFAS, are a class of about 15 000 (and growing) synthetic chemicals which are highly stable, very persistent, and widely used in countless consumer products.^1^ The adverse human health and wildlife effects of perfluorooctanoic acid (PFOA), perfluorooctane sulfonic acid (PFOS), and their derivatives, in particular, even at ng L^−1^ concentrations, has led the USEPA to establish a National Primary Drinking Water Regulation (NPDWR) of 4 ng L^−1^ for PFOA and PFOS individually and 10 ng L^−1^ for perfluorohexane sulfonic acid (PFHxS), perfluorononanoic acid (PFNA), and hexafluoropropylene oxide dimer acid (GenX).^2–5^ Conventional water resource recovery facilities (WRRFs) are not designed to eliminate PFAS and have been shown to discharge up to >1100 ng L^−1^ PFAS in the effluent,^6^ with up to 910 ng g^−1^ in biosolids,^7^ which limit land use application.^8,9^ Although PFAS have been effectively treated using electrochemical advanced oxidation or chemical redox reactions,^10^ they are highly resistant to biodegradation.^11–14^ Removal of PFAS from wastewater effluent has been attributed primarily to adsorption, resulting in their accumulation in wastewater sludge.^9,15^ The high stability of these PFAS is attributed to their strong C–F covalent bonds as well as their unique combination of hydrophilic headgroups and hydrophobic chains.

Variations in PFAS structures (*e.g.*, chain lengths, degrees of saturation, functional groups) significantly affect physicochemical properties that determine their distribution in wastewater mixed liquor/sludge as well as their potential for biodegradation.^15–17^ Polyfluorinated and unsaturated PFAS have been shown to have the highest susceptibility to biodegradation, with either a C–H bond at the α position or an unsaturated C=C bond being a key driver for bioavailability and defluorination.^18,19^ However, WRRFs are still dominated by long-chain perfluorinated legacy (PFOA, PFOS) and their replacement short-chain (GenX, PFBS) compounds that are extremely recalcitrant to both aerobic and anaerobic biodegradation,^11–14,19,20^ which target PFAS as a carbon source or electron acceptor, respectively. Successful degradation of PFOA and PFOS have been claimed only under highly specific environmental conditions and microbial species (*e.g.*, anaerobic iron co-metabolism with *Acidimicrobium* sp. strain A6 (ref. 21) or aerobic pure cultures that have used individual PFAS as the sole carbon source^22–25^). Recently, up to 90% biodegradation of PFOS by pure cultures of *L. portucalensis* strain F11 was reported, resulting in chain-shortening and defluorination.^26^ These studies suggest that despite the strong C–F bond in PFAS, specific bacteria and environmental conditions may have the ability to at least partially biodegrade these highly persistent chemicals, although degradation pathways have been unclear.

Besides the PFAS chemical structure, the source and composition of microbes present in biological systems greatly affect the degradation potential, pathways, and metabolites generated from different PFAS.^27–31^ For example, 6:2 fluorotelomer sulfonate (6:2 FTS) was shown to decrease by 36% under aerobic activated sludge,^32^ while aerobic sediment reduced concentrations by 80%, generating different transformation products in each case.^12^ Moreover, some studies have reported the impact of PFAS on microbial communities in different environmental matrices,^27–31^ although PFAS degradation in parallel with microbial community dynamics has rarely been reported. Some evidence from other hard to remove micropollutant and pharmaceutically active compounds (*e.g.* acetaminophen, ibuprofen and naproxen) have shown that slower growing heterotrophic bacteria at long solids retention times (SRTs) can more readily transform parent compounds from conventional activated sludge.^33^ The wide range of microbial communities present in wastewater treatment as well as our availability to shape community structure through operational parameters such as SRT, pH, and feeding composition may significantly alter the partitioning, fate, and transport of different PFAS.

Hence, there is a growing need to evaluate the biotransformation possibility of legacy and emerging PFAS that are dominant in WRRFs across microbial processes in environmentally relevant complex conditions (*e.g.* in the presence of other easily accessible carbon sources). Moreover, there is a need to better characterize parent and transformed PFAS removal due to both sorption and biotransformation in aqueous and biosolid phases, which are often reported as total removal or for the aqueous phase only (*e.g.*, ref. 23–25 and 34) to determine the ultimate fate and transport of PFAS in wastewater. To this end, this study comprehensively examined the potential biotransformation of five structurally variable legacy and replacement PFAS, including PFOA, PFOS, PFBS, GenX, and 6:2 FTS under both aerobic (activated sludge, nitrification sludge, mixed sludge) and anaerobic (KB-1® enrichment culture, anaerobic sludge) processes to examine the effect of microbial community dynamics and operational parameters which may drive differences in biotransformation. We specifically sought to examine the parent PFAS and formation of transformation products in both the aqueous and the solid phases in order to distinguish removal caused by adsorption and that caused by biotransformation. We also examined the microbial community responses over long-term operation of continuous wastewater reactors in response to different PFAS loading. Together, these results increase our understanding of PFAS biotransformation and partitioning across complex microbial community processes (*e.g.*, different SRT, feeding conditions) and environmental conditions (*e.g.*, anaerobic/aerobic processes).

## 2. Materials and methods

### 2.1 Chemicals

Standards of PFOA, PFOS, PFBS, 6:2 FTS and GenX were purchased from Fisher Scientific, Santa Cruz Biotechnologies, Sigma Aldrich, and Toronto Research Chemicals and mass-labelled PFAS were purchased from Wellington Laboratories. Analyte (abbreviation), molecular formula, CAS no., purity, molecular weight, LOD (ppb), LOQ (ppb), and structure are provided in Table S1.

### 2.2 Anaerobic culture design and operation

Long-term anaerobic treatment was performed in 200 mL basal medium using commercially available KB-1® enrichment culture (SiREM) or inoculum from anaerobic digester sludge (AnS) that shipped overnight on ice to UB, and immediately transferred to medium and incubation upon arrival. 160 mL basal medium^35^ (SI; section S1) was dispensed in 250 mL anaerobic flasks with caps and septum followed by sterilization at 121 °C. After cooling to room temperature, the flasks were flushed with nitrogen and spiked with 4 mL of sterile Ti(iii) NTA, 0.4 mL of sterile vitamin supplement (ATCC, vitamin supplement MD-VS) and 20 mL of PFAS stock solution (500 mg L^−1^ in water) to reach a final concentration of 50 mg L^−1^ PFAS in basal medium. Finally, the culture flask was inoculated with 15 mL (7.5% v/v) inoculate (AnS or KB-1®) to a total volume of 200 mL. 50 mL headspace was sparged with nitrogen gas.

For each of the culture flasks, lactate (500 mg L^−1^) was used as the electron donor with a single individual PFAS (50 mg L^−1^) compound introduced as the electron acceptor.^36^ Although the initial PFAS concentration of 50 mg L^−1^ exceeds environmental levels, this concentration was chosen in line with previous literature^18,21^ and allows for better identification of transformation products. Biotic and abiotic controls were performed in parallel to confirm culture viability and monitor cell growth using lactate and trichloroethylene (TCE, 50 mg L^−1^), and autoclaved inoculum (AnS or KB-1®) to measure abiotic PFAS removal for each compound (Table S3). While autoclaving may alter the matrix and therefore adsorption of abiotic controls, end-point biomass samples were also collected for all conditions to confirm results and analyze biomass for actual PFAS sorption. All culture flasks were run in duplicate and incubated at 34 °C for 90 days. Growth of KB-1® was confirmed with TCE degradation experiments (section S2; Fig. S1). 10 mL samples were collected every 7 days for the first 30 days and then every 14 days for the rest of the incubation period. pH was measured following centrifugation at 4000*g* for 30 minutes. The supernatant was collected for analyzing TCE, fluoride (F^−^), dissolved organic carbon (DOC), and PFAS and stored at −20 °C until analysis. The cell pellet was stored at −80 °C for DNA extraction.

### 2.3 Aerobic culture design and operation

Short-term (21 days) aerobic biotransformation assays were performed using the same five PFAS in 100 mL reaction volume as detailed above. Wastewater mixed communities were collected on the day of experiment from the return-activated sludge (RAS) from a local two-stage WWTP (Amherst, NY) for three initial inocula (activated [AS], nitrification [NS], and mixed sludge [MS], respectively; section S3). Inoculum for each assay was added at equal biomass concentration (total suspended solids [TSS]). Finally, each flask contained around 90 mL salt medium and 0.1 mL trace element solution (section S1) and spiked with PFAS stock solution (in methanol) that reached a final concentration of 2.00 g L^−1^ NaCl, 0.82 g L^−1^ MgCl_2_·6H_2_O, 0.12 g L^−1^ NH_4_Cl, 1.04 g L^−1^ KCl, 0.03 g L^−1^ CaCl_2_·2H_2_O, 0.40 g L^−1^ KH_2_PO_4_, 0.20 g L^−1^ MgSO_4_, and 0.05 g L^−1^ PFAS in 100 mL aqueous volume.^32,37^ As in anaerobic experiments, an abiotic (autoclaved) control was included using the MS culture for each PFAS (Table S4). All cultures were performed in triplicate in an incubator shaker (120 rpm in 25 °C) for 21 days. pH, dissolved oxygen (DO), and biomass samples (5 mL) were collected every 3 days for the 21 day incubation period. Briefly, samples were centrifuged at 13 000*g* for 15 minutes and the supernatant and cell pellet were stored for DNA and chemical analyses detailed above, with the addition of aqueous ammonium (NH_4_^+^). Endpoint biosolid samples were preserved at −40 °C for PFAS quantification. Both aqueous and biosolid fractions of initial inoculum NS (prior to spiking with PFAS) as well as PFAS stock solutions were subjected to PFAS quantification for parent compounds and targeted shorter-chain compounds (Table S6) to account for the background matrix.

### 2.4 Sample collection for PFAS analysis

For aqueous PFAS, aliquots of supernatant were collected from all anaerobic and aerobic long-term experiments during sampling for water quality and biomass. 20 μL aliquots of centrifuged supernatants were collected and diluted with 980 μL methanol. Finally, 30 μL of the previous mix was diluted to 200 μL with the starting mobile phase of a 45 : 55 ammonium acetate (5 mM) : methanol mixture, spiked with 150 μg L^−1^ mass-labelled internal standards (Table S1), and stored at −80 °C until analysis. At the end of the 21 day experiment, 50 mL samples were centrifuged to separate the endpoint biomass. Extraction of lyophilized biosolids were performed by solvent extraction following the method reported by Dickman *et al.*^38^ Extraction recoveries were evaluated by spiking biosolid samples with 1 mg L^−1^ MPFAC and 19 ES standard mix (Wellington Laboratories), and the percent recoveries of the targeted compounds were >65% except for PFBA (Table S7). The biosolid extraction (section S4) was performed for both inoculum NS (before spiking with PFAS) and incubated live and inactivated endpoint samples. The extract was analyzed by LCMS/MS for both parent and shorter-chain PFAS.

### 2.5 Identification and quantification of parent and transformed PFAS: liquid chromatography-tandem mass spectrometry (LC-MS/MS)

All PFAS quantification was performed using an Agilent 1200 HPLC instrument coupled to a Thermo Scientific™ TSQ Quantum Ultra triple quadrupole mass spectrometer (LC-MS/MS) operated in negative mode electrospray ionization (−ESI) with a spray voltage of +3000 V and capillary temperature of 300 °C. Nitrogen was used as the sheath gas (35 arbitrary units) and auxiliary gas (30 arbitrary units). Separation was achieved using a Restek Raptor C_18_ analytical column (2.7 μm particle size, 100 mm × 3 mm) and an aqueous mobile phase (A) of 5 mM ammonium acetate (pH 3.8), an organic mobile phase of methanol (B), and a flow rate of 0.27 mL min^−1^. A 27 minute gradient method was used to separate and analyze the PFAS starting with 55% B, ramping to 95% B over 13 minutes and held at 95% B for 9 minutes. The mobile phase was then returned to starting conditions of 55% (B) over 0.5 minutes and held for 5 minutes for equilibration before subsequent injection. In the case of Gen X analysis, the same instrumentation was used with some modification in the method. Separation was done in an isocratic elution with 40% mobile phase A and 60% mobile phase B. Capillary and vaporizer temperatures were both set at 100 °C, and sheath gas pressure and auxiliary gas pressure were set at 10 arbitrary units. PFAS quantification was performed using the isotopic dilution.^38^

A modified method was used to evaluate the possible formation of shorter-chain PFAS during the biodegradation assays. As the concentrations of transformed products were expected to be low, undiluted samples were used and spiked with mass-labelled internal standards. An identical LC-MS/MS chromatographic gradient was used; however, the chromatographic flow was diverted to waste until the end of the chromatographic method and not to the mass spectrometer before either PFOS, PFOA, or 6:2 FTS elutes.

### 2.6 Determination of fluoride ions

Fluoride ion concentrations in the aerobic experiments were measured using a fluoride ion selective electrode (ISE) (PerfectION comb F^−^, Mettler-Toledo) to determine the degree of defluorination. The ISE was calibrated using fluoride standards ranging from 10 μg L^−1^ to 100 mg L^−1^ (Fig. S2). The limit of detection (LOD) and limit of quantitation (LOQ) of the ISE were 28.4 and 31.8 μg L^−1^, respectively. Preparation of samples before ISE analysis includes the addition of 4 mL of TISAB II buffer to 4 mL of the samples. Only endpoint samples that underwent successful PFAS transformations were subjected to fluoride ion measurement.

### 2.7 DNA extraction, amplification, and sequencing

DNA was extracted using a DNeasy PowerSoil Pro Kit (Qiagen, Maryland, USA) following the manufacturer's instructions and eluted into 50 μl TE buffer. The extracted DNA was submitted at the Genomics and Bioinformatics Core at the University at Buffalo for sequencing. The sequencing targeted V4 region of the 16S rRNA gene for bacteria (forward [515F] = 5′-GTGYCAGCMGCCGCGGTAA-3′, reverse [806R] = 5′-GGACTACNVGGGTWTCTAAT-3′). The final mixture was sequenced with NextSeq 1000/2000 using a P1 XLEAP-SBS™ Reagent Kit (600 cycles, 2 × 300 paired end reads). All sequencing data can be found online at the NCBI Sequencing Read Archive (SRA) under BioProject accession number PRJNA1089582.

### 2.8 Sequence read processing and analysis

Sequencing reads were processed using the mothur v.1.48.0 software following the MiSeq SOP and a quality cutoff of 25.^39^ Following contig formation, sequences were then screened and aligned with the SILVA v138 SEED database followed by filtering, pre-clustering, and chimera removal using the VSEARCH tool. Sequences were classified with mothur-formatted RDP taxonomy and clustered in operational taxonomic units (OTUs) using a cutoff of 0.03. To further identify the taxonomic classification, representative sequences against each OTU were extracted using the get.oturep command and blasted in the NCBI database.

### 2.9 Statistical analysis

From the PFAS concentration data, a pseudo-first-order kinetics analysis was performed after the acclimation period (∼6 d), using ln(*C*/*C*_0_) *vs.* time to estimate the rate constant *k* (d^−1^) and *t*_1/2_ for each PFAS across the three sludge types (MS, AS, NS) (Fig. 2G–I and Tables S10 and S11). The estimated rate constants (presented as mean ± 95% CI) were then compared using analysis of variance (ANOVA) to determine the statistical significance across sludge types for PFOA, PFOS and 6:2 FTS at 95% confidence level (*α* = 0.05). Further *post hoc* analysis was conducted using Tukey's HSD test (*α* = 0.05) to evaluate pairwise differences among AS–MS, MS–NS and NS–AS for each compound individually. From the sequencing data, the alpha diversity indexes (Sob index, inverse Simpson index) and beta diversity indexes (Jaccard and Bray–Curtis dissimilarity) were calculated for diversity and distance among samples. PCoA analysis was performed with Bray–Curtis metrics to examine the relationship between samples of interest. The statistical significance of biological replicates with different PFAS exposures (PFOA, PFOS, and 6:2 FTS) using different sludge types was calculated using analysis of molecular variance (AMOVA, a nonparametric analog of traditional analysis of variance) with 95% confidence interval using Bray–Curtis metrics. Correlation between microbial community (top OTUs) and PFAS removal across each sludge type (MS, AS, NS) was analyzed using both Pearson and Spearman correlation (*p* ≤ 0.05).

## 3. Results and discussion

### 3.1 Removal of PFAS parent compounds

In this study, the total removal of the PFAS parent compound from the live aqueous solution was considered a combination of adsorption and microbial removal due to bacteria. Microbial removal here is attributed primarily to biotransformation (*i.e.*, chain-shortening or transformation of the parent compound) or bioaccumulation (*i.e.*, intracellular storage of the parent compound) following uptake by bacteria. Abiotic controls (bacteria inactivated by autoclaving) were used to quantify the percent removal due to adsorption only. The portion of total removal not attributed to adsorption (*i.e.*, total removal – abiotic removal) was attributed to microbial removal (section S5). At the end of experimentation, PFAS from biosolids and aqueous fractions were quantified to measure all parent compounds and targeted transformation products from both live experimental samples and abiotic controls (section 3.4; Fig. 4) and was shown to be due largely to biotransformation rather than bioaccumulation.

**Fig. 1:**
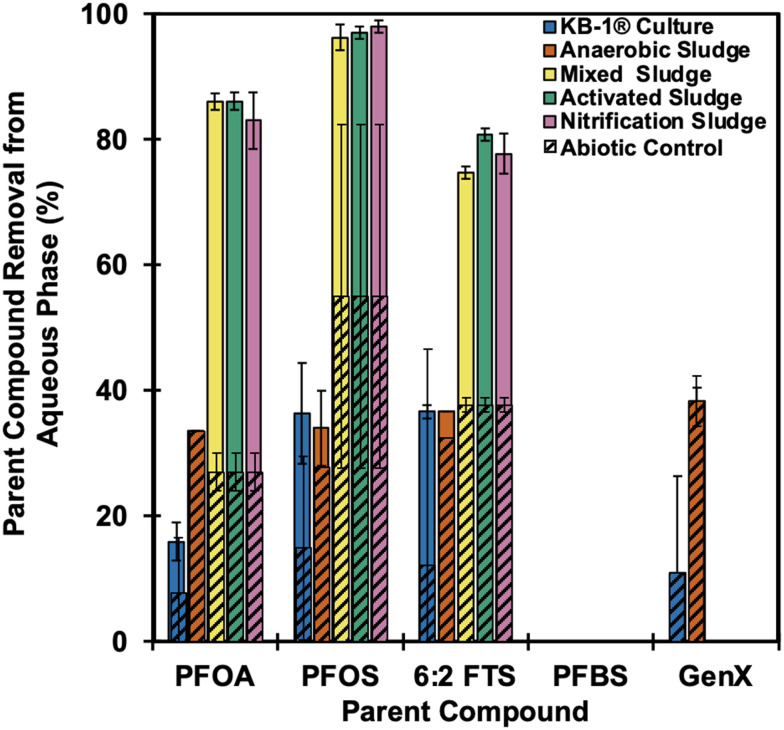
Maximum removal of 5 PFAS parent compounds from aqueous solution with 5 different microbial sources. Solid bars show total removal (combination of adsorption, bioaccumulation, and biotransformation) from live wastewater cultures, whereas hatched sections denote removal attributed to adsorption (quantified in abiotic controls). Aerobic cultures performed significantly better in microbially removing PFOA, PFOS and 6:2 FTS compared to anaerobic cultures. PFBS and GenX were not microbially removed except for some adsorption under aerobic or anaerobic conditions. All the aerobic experiments were run in triplicate, and all the anaerobic experiments were run in duplicate. Regardless, all the results are reported as the average ± standard deviation.

Among the five PFAS tested, PFOA, PFOS, and 6:2 FTS were removed (*i.e.*, 80–98% total removal) from the aqueous phase, with parent compound absolute microbial removal of 40–60% under aerobic conditions using wastewater microbial communities. Anaerobic microbial communities showed small amounts of sorption (10.9–38.3%) with no microbial removal of any of the PFAS tested (Fig. 1). All the aerobic experiments were run in triplicate, and all the anaerobic experiments were run in duplicate. Regardless, all the results are reported as the average ± standard deviation.

#### 3.1.1 Aerobic removal of PFAS

PFOA, PFOS, and 6:2 FTS were significantly removed across activated sludge (AS), nitrification sludge (NS), and mixed sludge (MS) cultures despite differences in initial microbial community structure. All three compounds achieved maximum removal of the parent compounds of 87.0 ± 1.4%, 97.0 ± 2.0%, and 80.0 ± 1.0%, respectively, in the aqueous phase (Fig. 1 and S10). Although the starting concentration (*C*_0_) was 50 mg L^−1^ for all PFAS with pH ∼6.5, the measured concentration at *t* = 0 for PFOA, PFOS and 6:2 FTS was 39.5 ± 5.5, 23.2 ± 1.7, and 50 ± 0.05 mg L^−1^, respectively, in abiotic controls, indicating very quick adsorption to sludge, particularly for PFOA and PFOS (Fig. S11). After 21 days, PFOA, PFOS and 6:2 FTS showed 26.9 ± 1.2%, 55.8 ± 1.4% and 37.7 ± 1.1% adsorption, resulting in 60.4 ± 1.6%, 40.7 ± 0.8% and 43.1 ± 1.0% microbial removal by live cultures, respectively. In contrast, previous studies have not shown significant microbial removal of PFOA and PFOS under aerobic conditions with mixed microbial species.^11^ Pure cultures (*e.g.*, *Pseudomonas* sp.), however, have shown some success in the degradation of parent compounds and generation of transformation products with efficiencies of 19–100%,^22–25^ although only one study reported defluorination.^24^ Importantly, these studies have used PFOA and PFOS as the sole carbon source; their biotransformation potential in the presence of other easily available carbon sources (as would be present in wastewater) has not been widely studied.^22^ However, for other recalcitrant compounds such as short-chain unsaturated carboxylic acids and micropollutants,^40,41^ the presence of additional growth substrate was shown to be necessary to maintain the enzyme-mediated metabolic activity. Despite previous findings that found 6:2 FTS to be more favorable for biodegradation than PFOA and PFOS (22–80% from diverse environmental matrices, including activated sludge,^32^ landfill leachate,^42^ and river sediment^12^), due to the absence of a C–F bond at the α and β positions, we found the contrary. The lower biotransformation of 6:2 FTS compared to PFOA and PFOS in this study might generate from the abundant sulfur source in the medium. Significantly, unlike carbon uptake, the absence of other sulfur sources may create a selective pressure that encourages the biotransformation of PFAS with sulfonic headgroups, and the highest reported degradation of 6:2 FTS (100%) was achieved by a pure culture *Gordonia* sp. strain NB4-1Y in the absence of other sulfur sources.^43^ In this study, wastewater and enrichment media had multiple carbon and sulfur sources in abundance. Similar to previous literature,^19,20^ no significant removals of PFBS and GenX were observed, indicating their non-bioavailability irrespective of the source and composition of microbes.

#### 3.1.2 Anaerobic removal of PFAS

During anaerobic treatment of PFAS, no net loss was observed beyond abiotic controls irrespective of inoculum source and structural variances. Both the KB-1® and the AnS had similar PFAS removal to the heat inactivated control, with removal due to physicochemical (*e.g.*, adsorption) rather than biological processes (Fig. S3), while biological controls degraded TCE completely within 7 days (Fig. S1). PFOA demonstrated a maximum of 20% and 35% removal by adsorption with KB-1® and AnS, respectively. For PFOS and 6:2 FTS, the measured concentrations were highly variable, with a maximum removal by adsorption of 25% PFOS and 20% 6:2 FTS in the 90 day incubation period with KB-1®. As expected, PFBS did not undergo biotransformation or adsorption, which can be attributed to the low hydrophobicity (shorter hydrophobic C–F tail) and high polarity. While perfluorinated compounds, including PFOA and PFOS, have been hypothesized to be used as an electron acceptor for reductive dehalogenation,^11,34^ their negative redox potential (−0.450 V) restricts their coupling to an oxidizable substrate that can generate sufficient energy.^44^ As with all perfluorinated compounds, the lack of unsaturated C–F bonds at the α position and C=C bonds make PFOA, PFOS, and PFBS generally recalcitrant to anaerobic degradation.^13,18,20^ In case of 6:2 FTS and GenX specifically, microbial desulfonation and ether bridge splitting are the rate-limiting steps.^12,19,45^ Thus far, the only successful anaerobic degradation has been shown in the presence of a cometabolite (*i.e.*, *Acidimicrobium* strain A6 and the Feammox reaction^34^), which resulted in 50% reduction in PFOA and 47% reduction in PFOS.^34^ Reduction under anaerobic co-metabolism has also been shown in simulated sewage using low concentrations of PFOS (100 μg L^−1^; 24% removal) with formation of transformation by-products, but defluorination was not reported.^46^ In addition to making PFAS reduction more energetically favorable through co-metabolism in the presence of another terminal electron acceptor, it may only be achievable with an enzymatically catalytic system for selected unsaturated compounds.

### 3.2 Aerobic microbial removal of PFAS over time

**Fig. 2:**
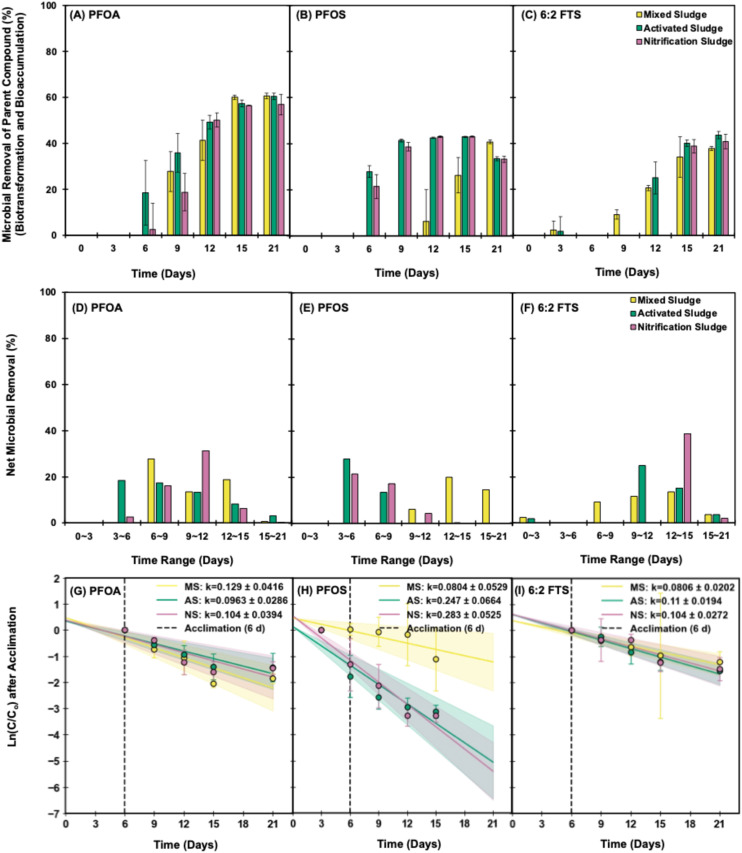
Total aerobic microbial removal (total removal – adsorption; aqueous phase) of (A) PFOA, (B) PFOS and (C) 6:2 FTS with MS (yellow), AS (green) and NS (lavender) cultures over 21 days. Bacteria with slower growth kinetics were associated with the removal of long-chain PFAS, with net microbial removal for (D) PFOA, (E) PFOS and (F) 6:2 FTS peaking between 3 and 12 days of reactor operation. A pseudo-first-order kinetics analysis after acclimation (∼6 d) was performed using ln(*C*/*C*_0_) *vs.* time to estimate the rate constant *k* (d^−1^) for (G) PFOA, (H) PFOS and (I) 6:2 FTS. The rate constants were significantly different for PFOA (*ρ* = 0.028), PFOS (*ρ* = 0.0004) and 6:2 FTS (*ρ* = 0.018) at a 95% confidence level (*α* = 0.05) across MS/AS/NS.

After an acclimation period (∼6 days) and community shift, PFOA, PFOS and 6:2 FTS were rapidly removed due to bacterial growth under all aerobic sludges within 21 days (Fig. 2A–C). PFOA was reduced by 6.34–27.7% in each time range from 6 to 12 days (Fig. 2D) and achieved a maximum of 56.4–60.6% microbial removal in all sludge types occurring within 15 days of incubation (Fig. 2A). PFOS removal occurred faster, with 13.4–27.8% reduction in each time range from 3 to 9 days (Fig. 2E), resulting in 43.1% and 42.5% microbial removal using activated and nitrification sludge, respectively, occurring within 9 days (Fig. 2B). In contrast, the maximum microbial removal of 6:2 FTS was only 37–43% after 15 days (Fig. 2C). A pseudo-first-order kinetics analysis after acclimation (∼6 days), using ln(*C*/*C*_0_) *vs.* time (*t* >6 d; *C* corrected by the abiotic control), was performed to estimate the rate constant *k* (d^−1^) and *t*_1/2_ for each PFAS and sludge type (Fig. 2G–I and Tables S10 and S11). For each PFAS, there were three replicates per sludge type, and the plot shows the mean ± 95% CI. This was further analyzed using ANOVA, which showed that the rate constants were significantly different for PFOA (*ρ* = 0.028), PFOS (*ρ* = 0.0004) and 6:2 FTS (*ρ* = 0.018) at a 95% confidence level (*α* = 0.05) across MS/AS/NS (Table S12). Further analysis using Tukey's HSD test (*α* = 0.05) indicated that for PFOA, the rate constants were significantly different between AS and MS only, whereas for PFOS and 6:2 FTS, the rate constants were significantly different between AS–MS and NS–MS (Table S13).

While initial wastewater communities did not remove PFAS except by adsorption, longer SRTs that allow the shift of initial inoculum and growth of slow-growing heterotrophs may result in communities more capable of tolerating and removing toxic PFAS (Fig. 2 and 5). Longer SRTs have been correlated with higher biotransformation of numerous micropollutants and pharmaceutically active compounds, especially those that undergo oxidative reactions.^33,41,47,48^ Higher SRT not only promotes the growth of slow-growing organisms but also changes enzyme expression at the molecular level with decreasing food-to-microbe ratio.^41^ While easily biodegradable carbons are rapidly consumed, higher SRT has been shown to allow the increased expression of enzymes like cytochrome P450, which allow the utilization of a wider range of substrates.^41^ The dominant mechanism of PFAS removal from wastewater is shown to be sorption through hydrophobic interaction,^27,49,50^ possibly due to the low retention time of biomass and transformation of long-chain unsaturated precursors to short-chain saturated PFAAs through weaker C–C cleavage.^51^ However, this study shows that given sufficient acclimation time, removal of saturated long-chain PFAS through microbial removal may occur in parallel with biosolid sorption.

### 3.3 Transformation and partitioning of PFAS parent compounds

**Fig. 3:**
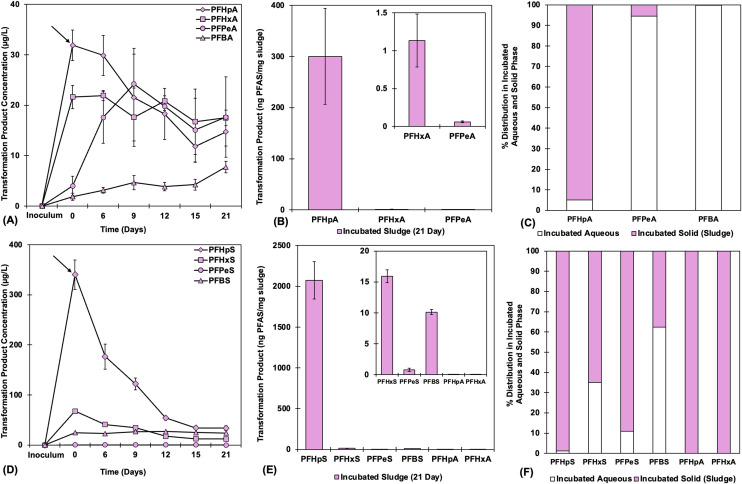
Transformation of PFOA and PFOS into shorter-chain C_4_–C_7_ compounds under NS conditions in (A and D) aqueous and (B and E) solid phase. The arrow shows the concentration right after spiking with the parent compound. Significant generation of PFPeA and PFBA in the aqueous phase is observed from PFOA degradation over 21 days. Initial sludge had no targeted carboxylates with negligible (0.04–0.15 ng mg^−1^) targeted sulfonates. However, after 21 days there was significant generation of both shorter-chain carboxylates and sulfonates with C_7_ homologues identified in the highest amount. Partitioning to sludge increases with chain length, as seen in percent distribution from (C) PFOA and (F) PFOS.

**Fig. 4:**
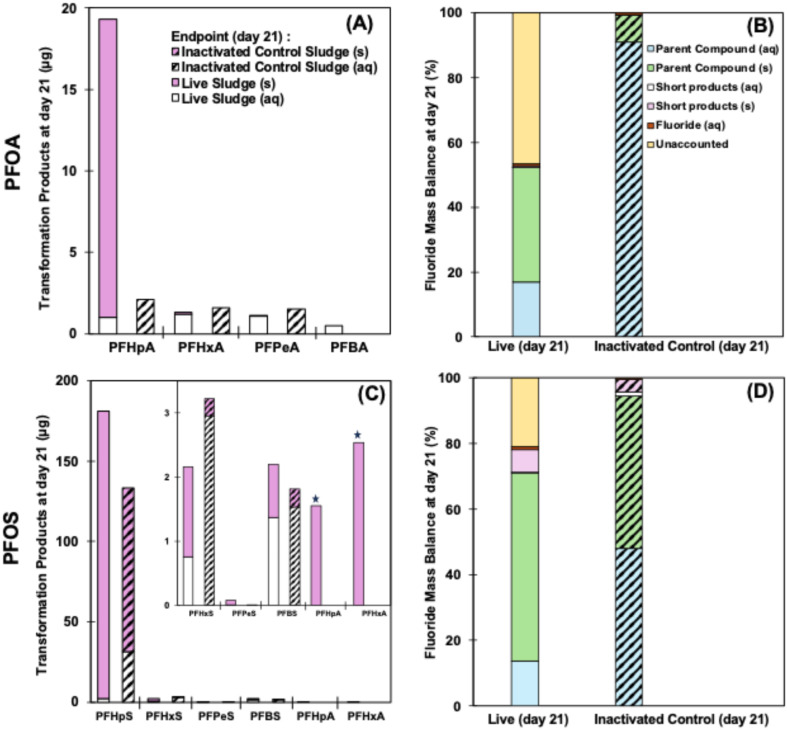
Transformation of PFOA (A) and PFOS (C) into shorter-chain C_4_–C_7_ compounds and fluoride mass balance of PFOA (B) and PFOS (D) under nitrification conditions in both aqueous and solid phases for both live (solid bar) and inactivated control (hatched bar) at the end of 21 days incubation period. C_4_–C_7_ homologues were 1.28–14.5-fold abundant in live sludge than control sludge with the presence of some unique compounds (A: PFBA), (C: PFPeS, PFHpA, PFHxA) only in live sludge. Star (C) denotes amplification of values by 1000 for better visualization. For endpoint fluoride mass balance (B and D), the concentration of parent compounds and targeted transformation products in aqueous and solid phases, and fluoride, in both live and autoclaved killed control sludge, were considered. Overall, 46.7% PFOA and 21% PFOS were unaccounted for and attributed to non-target transformation products. There were no unaccounted-for products in the inactivated controls.

In addition to the measurement of the PFAS parent compound, the generation of targeted biotransformation by-products and the release of F^−^ ions were considered key indicators of successful parent compound biotransformation. Aqueous concentrations of target biotransformation products were quantified at every sampling point for all sludge types, and endpoint biosolid samples (nitrification sludge) were extracted to quantify bioaccumulation and biotransformation from microbial biomass. Targeted short-chain transformation products (C_4_–C_7_ homologues) were detected in both aqueous and biosolid phases (Fig. 3, NS, and S7) from PFOA and PFOS microbial treatments. There were no detected targeted transformation products for 6:2 FTS except constant low detection of 4:2 FTS, likely from stock impurity (Fig. S8). In the aqueous phase of PFOA-treated active samples, PFPeA and PFBA were generated (days 6–15) across all sludge conditions, with a maximum of 24.2 ± 7.07 μg L^−1^ and 7.75 ± 1.14 μg L^−1^ for PFPeA and PFBA, respectively, in NS (Fig. 3A). While PFPeA reached a maximum concentration at 9 days, PFBA continued to increase until day 21, increasing from 4.66 ± 1.40 μg L^−1^ to 7.75 ± 1.14 μg L^−1^, indicating the possibility of further transformation of PFPeA to PFBA, even after PFOA transformation stopped. The presence of PFBA was identified only in live aqueous samples and was absent in aqueous and sludge controls (Fig. 4A). The highest fraction of transformation products from PFOA was PFHpA that adsorbed to solids (Fig. 3B), with the concentration of PFHpA of 300 ± 93.8 ng mg^−1^, which was absent in the autoclaved control sludge (Fig. 4A). This higher adsorption of longer transformation products is consistent with previous observations, in which the partitioning potential increases with increasing chain length due to the strong hydrophobic effect of the C–F chain (Fig. 3C).^15–17^ While nontarget intermediate PFAS metabolites and volatile end products were not measured, the identified transformation products are consistent with past research. Previously, one study among two successful aerobic biodegradation studies of PFOA^22,25^ has shown similar transformation products (PFPeA, PFHxA, PFHpA) but none has reported defluorination. Although a transformation pathway was not proposed in this study, the identified transformation products suggest that the C–C bond was oxidized and stepwise the –CF_2_– group was removed to generate the shorter-chain products. The identified transformation products PFHpA, PFHxA, PFPeA and PFBA are similar to the transformation products that were previously recorded in anaerobic studies.^34,52^

On the other hand, significant generation of aqueous short-chain products were not seen in PFOS cultures (Fig. 3D). PFHpS, PFHxS, PFPeS and PFBS increased ∼1.1–1.5× their initial presence and continued to decrease along with the PFOS transformation in MS (Fig. S7). However, PFOS transformation products adsorbed to biomass and were found in the solids fraction at the end of the experiment (Fig. 3E and F). The highest fraction came from PFHpS (2073 ± 226 ng mg^−1^), while the concentration of PFHxS, PFPeS and PFBS was 2073 ± 226 ng mg^−1^, 15.9 ± 1.05 ng mg^−1^, 0.80 ± 0.28 ng mg^−1^, and 10.1 ± 0.40 ng mg^−1^, respectively, with trace amounts of PFHpA: 0.02 ± 0.00 ng mg^−1^ and PFHxA: 0.03 ± 0.01 ng mg^−1^. The presence of shorter-chain carboxylates was observed only in live samples (Fig. 4B), which indicates that PFOS was transformed to shorter-chain sulfonates and carboxylates, but due to their higher hydrophobicity and number of acidic sulfonic groups,^15–17^ the majority of the shorter-chain compounds were adsorbed on biosolids. Limited studies with PFOS biodegradation have identified PFHxS, PFBS, PFHpA and PFHxA,^23–25^ whereas two studies reported defluorination^24,26^ during aerobic transformation. In this study, not only the shorter-chain sulfonates (PFHpS, PFHxS, PFPeS and PFBS) but also the shorter-chain carboxylates (PFHpA and PFHxA) were detected, with PFHpS and PFPeS identified from the aerobic biodegradation of PFOS with appropriate controls. The shorter-chain sulfonates were most likely generated by partial cleavage of the C–C bond through decarboxylation and defluorination, whereas for generating the shorter-chain carboxylates, the sulfonic headgroup was removed and carboxylated followed by subsequent fluoride removal. Other than these targeted compounds, another study has reported the formation of defluorinated intermediates from PFOS by using ultra-performance liquid chromatography with ion mobility separation coupled to a time-of-flight mass spectrometer, which strengthens the idea of aerobic biotransformation of PFOS,^26^ although the specific pathway is still unknown.

Overall, for both PFOA and PFOS, the C_7_ homologues were the highest identified transformation products, considering both aqueous and biosolid phases. On the other hand, 6:2 FTS has reportedly transformed into 6:2 FTOH, 6:2 FTCA, 6:2 FTUCA, 5:3 FTCA, PFHpA, PFHxA, PFPeA, PFBA, 5:3 ketone, and 5:2 FTOH in a few studies^12,32,42,43,53^ but defluorination was reported only once.^42^ Here, small amounts of microbial defluorination were successfully recorded from 6:2 FTS despite the absence of targeted transformed products (C_4_–C_7_ carboxylates and sulfonates only), which could be due to the presence of sulfate inhibiting desulfonation or lack of sufficient monooxygenases to catalyze the reaction,^54,55^ or other unidentified by-products that were not included in the target analysis.

Detection of net F^−^ (aqueous phase, subtracting free F^−^ detected in abiotic controls; Fig. S9) shows that small amounts of mineralization did occur, with net F^−^ of 36.8–134 μg L^−1^ for PFOA, 16.5–77.9 μg L^−1^ for PFOS, and 6.38–63 μg L^−1^ for 6:2 FTS, depending on sludge type. The presence of small but consistent detection of F^−^ in the system suggests the partial defluorination of parent compounds that followed mostly chain-shortening transformation pathways. However, the amount of F^−^ detection did not match the identified transformation products, making it likely that non-target compounds were formed. Some defluorination may also have been underestimated, as generated F^−^ may have formed volatile HF.

### 3.4 Overall mass balance

The total mass balance for each culture was calculated using end point (day 21) aqueous and solid phases for parent compounds and targeted transformation products as well as aqueous fluoride in both live nitrification sludge and inactivated control sludge. Ultimately, 52.13% PFOA and 71.04% PFOS in the live cultures and 100% PFOA and 100% PFOS in control cultures remain unaltered. The live samples indicated a higher proportion of adsorption-associated parent compound (13.35–17.55%) in the final solid partition than abiotic controls for PFOA and PFOS, respectively. This may have been caused by biotic accumulation in microbial cells or differences in pH between abiotic/biotic samples, the latter of which underwent nitrification. Although long-chain PFAS in this study were transformed in the range of 47.87% and 28.96% for PFOA and PFOS, respectively, the quantified transformation products and F^−^ do not represent a significant fraction (1.16–7.98%) of the total F^−^ mass balance. Even so, there is still 46.7% PFOA (Fig. 4C and Table S8) and 21% PFOS (Fig. 4D and Table S8), unaccounted for in live sludge, however, 100% mass balance was achieved for both PFOA (Fig. 4C and Table S8) and PFOS (Fig. 4D and Table S8) in inactivated controls. The unaccounted-for portion of PFOA and PFOS fluoride mass balance in live samples may have biotransformed to nontarget products or undergone chain-shortening that formed relatively volatile end products such as TFA and other 2–3 chain compounds, which were not covered by this study. The analysis of TFA and other ultra-short chain PFAS requires a separate methodology and is difficult to achieve, hence, these compounds are rarely reported in degradation studies.^56^ This warrants more comprehensive examination for parent and nontarget transformation products in all phases, including the gaseous phase, to achieve a better mass balance. The generation of PFOA and PFOS transformation products observed in this study are attributed to the biotransformation of spiked parent compounds under aerobic microbial communities. Although some of the targeted C_4_–C_7_ homologues were also detected in both PFOA and PFOS abiotic controls, they were 1.21–14.5-fold smaller (except for C_6_ homologues) than live samples (Fig. 4A and C) and attributed to low levels of PFAS stock impurities. The abundance of transformation products in live and control samples was statistically different (*p* < 0.01 for PFOA; *p* = 0.038 for PFOS). Additionally, no indication of precursor degradation, which would have resulted in the presence of the same short-chain compounds across experimental conditions, was observed. To date, no biotransformation study has conclusively accounted for 100% F^−^ mass balance. While this study also fell short of full balance, it highlights the often-overlooked partitioning of PFAS and their transformation products between live and control sludge—an area warranting further investigation—to understand the fate and transport of these compounds.

### 3.5 Microbial community dynamics

**Fig. 5:**
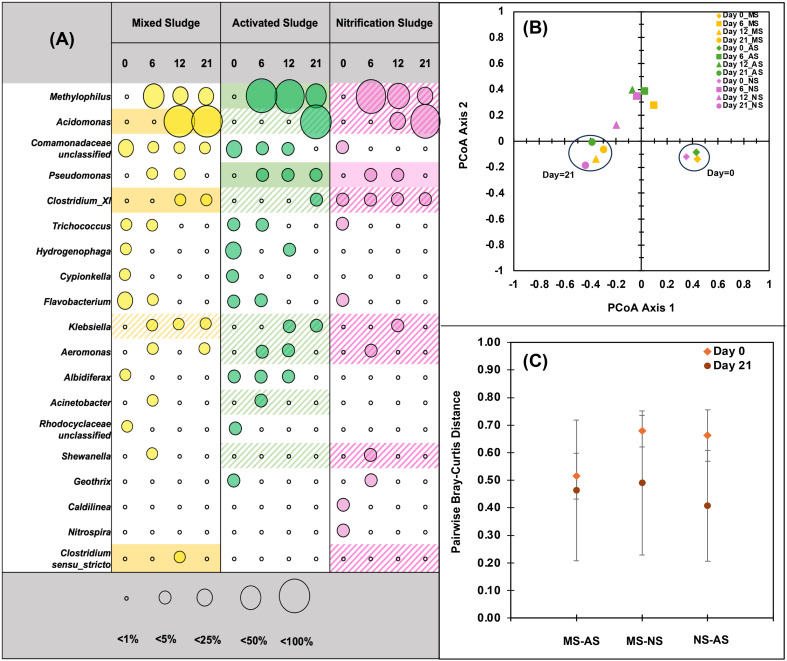
(A) Mean relative abundance of the top 1% and 0.5% OTUs from MS/AS and NS cultures (totaling 19 OTUs) over 21 days. Each circle represents the average microbial community observed under PFOA, PFOS, and 6:2 FTS at each sampling point. Hatched areas represent microbes that were positively correlated with degradation. Shaded areas indicate significant positive correlations (Pearson; *p* < 0.05). (B) PCoA visualization using Bray–Curtis dissimilarity metrics showed that community shifted over time – starting at day 0 (diamonds), the community completely shifted towards a steady state at day 21 (circles) across all sludge conditions (yellow – MS, green – AS and lavender – NS). (C) Pairwise distances decreased between MS–AS, MS–NS and NS–AS at days 0 and 21, indicating that under PFAS stress and operational conditions, microbial communities shifted and converged towards a similar community.

Initial wastewater inocula were composed of typical, highly diverse bacteria present in activated sludge systems,^57^ including Proteobacteria (41.3 ± 12.0%), Bacteroidetes (15.7 ± 3.54%), Firmicutes (8.21 ± 1.37%), and Actinobacteria (10.1 ± 1.46%), with the nitrification sludge also having Chloroflexi (6.44%), Planctomycetes (9.62%), Acidobacteria (4.29%), Verrucomicrobia (3.64%), and Nitrospira (1.36%) among other phyla (Fig. S5). At the genus level, the inocula in both AS and MS were dominated by *Comamonadaceae* gen. (4.85–14.5%), *Trichococcus* (2.52–4.71%), *Hydrogenophaga* (2.62–7.34%), *Cypionkella* (2.45–5.31%) and *Flavobacterium* (6.21–8.96%) (Fig. 5A and S4), with NS having higher diversity. While initial starting communities displayed high diversity (*S*_sobs_ = 3246, 1833, and 2306; *D*_INVSIMPSON_ = 47.8, 50.4, and 189.5 for MS, AS, and NS, respectively), communities quickly (6 days) shifted under PFAS loading and environmental conditions with a significant reduction in diversity after 21 days (*S*_sobs_ = 862.5, 649, and 477; *D*_INVSIMPSON_ = 3.8, 2.8, and 1.9, respectively), as expected when applying selective pressures (*e.g.* PFOA) imposed under laboratory conditions.^58^ Indeed, environmental and laboratory conditions (*e.g.*, medium, pH, and soluble carbon type) appeared to have the largest impact on the microbial community structure. There was no significant difference between biological replicates with different PFAS exposures (PFOA, PFOS, and 6:2 FTS) using different sludge types (Table S5), and dominant genera include those we have previously seen in non-PFAS biological controls (*e.g.*, *Pseudomonas*, *Methylophilus*). Despite this, specific genera correlated to PFAS removal rates (Fig. 5A and S6) and PFAS removal was only observed after acclimation and the initial community changes. Specific genera (*e.g.*, *Pseudomonas*) seen here have been previously reported to proliferate and survive when subjected to PFAS stress.^59–61^

Because there was no significant difference between biological replicates, the microbial community for each sampling point was averaged across PFOA, PFOS and 6:2 FTS (Fig. 5A). The three significantly different microbial inocula (AMOVA, *ρ* = 0.002 for AS–MS, *ρ* = 0.001 for AS–NS and *ρ* < 0.001 for NS–MS) with different rates of community shift, ultimately converged to the same community with no significant difference after 21 days (AMOVA, *ρ* > 0.05) (Fig. 5A and B). At the end of the study, all microbial communities were dominated by Proteobacteria (90.7 ± 3.29%) which is in agreement with previous studies,^29,59,62,63^ possibly due to the interaction of PFAS with the phospholipid outer membrane present in the nominally Gram-negative bacteria.^64^ At the genus level, only 5 of 19 communities comprising the initial microbial community abundance (Fig. 5B; OTUs >1%) were prevalent with significant genera belonging to *Acidomonas* (56.9 ± 29.6%), *Methylophilus* (22.7 ± 13.9%), *Clostridium_XI* (2.79 ± 1.77), *Klebsiella* (1.18 ± 1.76%) and *Pseudomonas* (0.87 ± 0.93%). PCoA visualization using Bray–Curtis dissimilarity metrics showed that microbial communities across all cultures and PFAS shifted and converged from the initial community (Fig. 5B). The dominance and convergence towards a few genera across all reactors can be further verified by the low diversity indices as well as pairwise Bray–Curtis distances at the end of the study period (Fig. 5C). The three communities were different initially (pairwise Bray–Curtis distances: 0.52 ± 0.08, 0.68 ± 0.06, 0.66 ± 0.09 for MS–AS, MS–NS and NS–AS, respectively); however, after 21 days of incubation the differences became less prominent (pairwise Bray–Curtis distances 0.46 ± 0.25, 0.49 ± 0.26, 0.41 ± 0.20, respectively).

Specific genera, including *Pseudomonas* (0.21 *vs.* 3.3%), *Acinetobacter* (0.59 *vs.* 2.45%), *Clostridium* (1.03 *vs.* 3.3%), *Klebsiella* (0.14% *vs.* 1.77%), and *Methylophilus* and *Acidomonas*, are positively correlated to the degradation of long-chain PFAS (Fig. S6). The increase of *Pseudomonas* (0.21% *vs.* 3.3%), in particular, was significantly positively correlated across all reactors in this study during days 6–12 when net degradations were highest. Recently, multiple pure culture *Pseudomonas* strains (*e.g.*, *Pseudomonas aeruginosa* strain HJ4, *Pseudomonas plecoglossicida* 2.4-D) have been specifically found to degrade PFOA, PFOS and 6:2 FTS under aerobic conditions.^22–25,65^*Pseudomonas* is also found to proliferate under PFOA and PFOS stress in various environmental matrices (surface water, rural drinking water, and activated sludge)^59–61^ and has been shown to metabolize a wide range of xenobiotic contaminants because of the ability to release biosurfactants that enhance cell permeability and increase the solubility of hydrophobic contaminants. In addition to *Pseudomonas*, multiple studies have reported a positive correlation of different PFAS with *Acinetobacter* (*e.g.*, ref. 59, 60 and 66), *Klebsiella*,^60^ and *Clostridium*.^67^*Acinetobacter* can also produce biosurfactants to survive in a complicated environment and degrade halogenated and organic compounds (PCE, TCE, BTEX, diesel and oil).^66^*Acinetobacter* was recently shown to biodegrade 27% perfluorooctane sulfonamide (PFOSA) by its localized extracellular enzymes under 12 hours through selectively cleaving the C–C and C–F bonds over C–S bonds in a multi-step pathway.^68^ Interestingly, *Pseudomonas* and *Acinetobacter* can coexist under cometabolic conditions during the dehalogenation of chlorinated compounds.^66^*Clostridium* has recently been found to have the capability to defluorinate unsaturated carboxylic acids of different chain lengths such as PFUPA.^67^*Clostridium* contains electron bifurcating enoyl-CoA reductases, homologues of CarCDE, and is known to reduce short-chain acyl-CoA and other short-chain substrates.^67^ As the experimental setup contains substrates other than PFAS only, cometabolic degradation using oxygenase enzymes and desulfonation using alkane sulfonate monooxygenase genes might have been associated with PFAS degradation. Previously, polyfluorinated PFAS containing sulfonic groups such as H-PFOS, 6:2 FTAB, and 6:2 FTSA have undergone desulfonation using *Pseudomonas* sp. strain D2,^55^*Gordonia* sp. strain NB4-1Y,^43^ and *Rhodococcus jostii* RHA,^69^ respectively. Bacterial degradation starts by attacking the α-C or the C–S bond and uses sulfonated PFAS as a source of sulfur with an upregulation of alkane sulfonate monooxygenase gene. On the other hand, oxygenase enzymes are widely known to catalyze compounds other than their primary substrates. During the transformation of 6:2 FtAoS, oxygenase enzymes (toluene dioxygenase, cytochrome P450, and alkane monooxygenase) were correlated.^70^ In our study, methanol was present as an organic donor in addition to the organic carbon present in the wastewater media, and methanol-utilizing bacteria were a dominant fraction of the microbial community. Previous studies have reported that these bacteria can increase as a result of the removal of other single unsaturated carbon compounds or more complex compounds like benzophenone-3.^71,72^ Recent studies have found that under the selective pressure of PFOA, *Methylophilus* also increases with PFOA concentration.^59,62^ These studies do not report whether methanol was present in PFOA stock, which might also lead to the observed increase rather than the impact of PFOA only, which requires further investigation. However, a separate study demonstrated that methylotrophic bacteria (*Methylocaldum*, *Methylobacillus*, *Methylobacterium*) increase in the presence of 6:2 FTS-spiked methanol compared to non-spiked methanol, suggesting they have higher tolerance against 6:2 FTS.^37^ These results underscore the importance of the selective pressure of PFAS and environmental conditions in shaping the microbial community that can tolerate long-chain PFAS despite having different inocula. Further research into the effects that specific operational parameters (*e.g.*, pH, carbon source) have in controlling community structure and removal under PFAS loading should be performed.

## 4. Conclusions

This study demonstrates the ability of wastewater microbial communities to differentially adsorb and biotransform long-chain legacy PFAS across microbial processes. However, short-chain “replacement compounds” PFBS and GenX were not transformed under either aerobic or anaerobic conditions, and short-chain PFAS have been shown to be particularly stable as well as mobile in the aqueous phase. The effect of decreasing perfluorinated chain length on bioavailability should be considered while designing new replacement compounds to reduce the global PFAS footprint. Here, the identification and partitioning of transformed shorter-chain products in both aqueous and solid phases provide a better understanding of the fate, biotransformation and bioaccumulation of these long-chain compounds in the downstream effluent and biosolids of WRRFs. Given sufficient time, slow-growing heterotrophs may tolerate and biotransform recalcitrant PFAS under cometabolic activity involving multiple species, resulting in different biotransformation products and removal rates than those due to adsorption only.

## Conflicts of interest

There are no conflicts to declare.

## Note added after first publication

This article replaces the version published on 17th November 2025. The details for Ref. 65 have been corrected.

## Supplementary Material

EW-012-D5EW00528K-s001

## Data Availability

The data supporting this article have been included as part of the supplementary information (SI). All sequencing data can be found online at the NCBI Sequencing Read Archive (SRA) under BioProject accession number PRJNA1089582. Supplementary information is available. See DOI: https://doi.org/10.1039/d5ew00528k.
