# Supplementary material for: Biotransformation and partitioning of structurally different PFAS by wastewater microbial consortia
Source: Environ Sci (Camb). 2025 Oct 27;12(1):227–41. doi: 10.1039/d5ew00528k (PMC12652306; doi:10.1039/d5ew00528k)
Supplement: EW-012-D5EW00528K-s001 [file EW-012-D5EW00528K-s001.pdf]

Supplementary Information (SI) for Environmental Science: Water Research & Technology.
This journal is © The Royal Society of Chemistry 2025

Supporting Information for:
Biotransformation and Partitioning of Structurally Different PFAS by Wastewater Microbial Consortia

1. Supporting Text

Section S1: Anaerobic Basal Media Recipe

Anaerobic basal media was prepared as needed using the following recipe to produce 1 liter. 954.09 mL of DI water were added to a 1 L glass bottle. 40 mL salt solution, 2 mL trace element solution, 2 mL Se/Wo solution, 0.25 mL Resazurin solution, and 1.66 mL sodium lactate were added. This mixture was brought to a boil, then reduced to room temperature under N_2. 48.036 mg of Na_2S (2 mmol) and 24.2308 mg of L-cysteine (2 mmol) were also added, and the bottle was thoroughly mixed. The bottle was then autoclaved at 121°C, then allowed to cool to room temperature once again. The bottle was tightly sealed and used for experiment as soon as possible. Media was dispensed anaerobically under 100% nitrogen. Recipes for individual solutions follow below.

The salt solution was prepared in 1000 mL of DI water using 50 g NaCl, 20.5 g $MgCl_2•6H_2O$, 13.5 g NH_4Cl, 26 g KCl, 0.75 g $CaCl_2•2H_2O$, and 10 g KH_2PO_4. This solution was stored at room temperature.

The trace element solution was prepared in 1000 mL of DI water using 10 mL HCl (25% w/w), 1.5 g $FeCl_2•4H_20$, 6.0 mg H_3BO_3, 190 mg $CoCl_2•6H_2O$, 100 mg $MnCl_2•4H_2O$, 70 mg $ZnCl_2$, 36 mg $Na_2MoO_4•2H_2O$, 24 mg $NiCl_2•6H_2O$, and 2 mg $CuCl_2•2H_2O$. The solution was stored in dark conditions at room temperature.

The Se/Wo solution was prepared in 1000 mL of DI water using 0.5 g NaOH, 6.0 mg $Na_2SeO_3•5H_2O$, and 8 mg $Na_2WO_4•2H_2O$. This solution was stored in dark conditions at room temperature.The Resazurin solution was prepared in 100 mL and 0.1 g of Resazurin.Ti(III)NTA was prepped to 25mM and also filter sterilized.

Section S2: Analytical Measurement of TCE by GC-ECD

All TCE samples were analyzed using a gas chromatograph coupled with an electron capture detector (GC-ECD, Agilent 7890B-^{63}Ni ECD) with an HP-5 (30 m x 320 µm x 0.25 µm) column using a modified version of previous work.[1] ECD temperature was 290°C with helium carrier gas and a mixture of methane and argon as makeup gas with a flow rate of 18.8 mL/min. The oven program was set to start at 30 °C, hold for 10 minutes, then ramp up at 50 °C per minute until reaching 200 °C. Once at the final temperature, the oven would stay here for 4 minutes, for a total run time of 17.4 minutes. The column pressure was 5.7 psi with a flow of 1.2 mL/min. High concentrated TCE from culture flasks was diluted in methanol in first step. A second dilution step was performed in an analytical vial in tert butyl methyl ether (MTBE) to get samples in the linear range of the detector. Tetra chloro ethylene (PCE) was used as an internal standard. Calibrations

were completed at the beginning of run days, consisting of 5 concentrations, in duplicate. Concentrations were chosen to represent the range of concentrations in sample vials after dilutions. A calibration stock was created using the methanol stock solution and diluting into MTBE.

Section S3: Town of Amherst Wastewater Treatment Facility

Town of Amherst wastewater treatment facility, NY, USA treats an average of 24 million gallons of wastewater per day. The facility provides a two-stage aerobic treatment process where in the first stage secondary treatment it removes organic matter (reduces 75% of the influent BOD) and in the second stage tertiary treatment it works on nitrification (reduces the BOD to 6 mg/L). Each of the stage has separate sludge settlers. The first stage has a HRT of 1h and SRT of 8±4 d and mostly generates heterotrophic activated sludge (RAS 1), where the second stage has a HRT of 2h and SRT of 35±14 d and mostly generates nitrifying sludge (RAS 2). The TSS for the return activated sludge for stage 1 and 2 are 13090 mg/L and 5805 mg/L respectively.

	TSS (mg/L)	Volume added (mL)
RAS 1 (AS)	13090	7.2
RAS 2 (NS)	5805	16.3
Mixed (MS)	9447	10

Section S4: Biosolid Extraction Procedure (adapted from Dickman & Aga, 2022)

Stored biosolid samples were weighed into PP tubes (50 mL) and then fortified with 25 µL of the 1 mg/L MPFAC, 19 ES standard mix . A series of ultrasonication solvent extractions were used, starting with solvent A aqueous acetic acid (1% v/v) (7.5 mL) and then solvent B 90:10 (v/v) methanol: aqueous acetic acid (1% v/v) (1.7 mL), repeating for a total of 6 cycles of sonication. In each cycle, samples were vortexed (30 s), sonicated (15 min, 60 ºC), and centrifuged (10 min, 3700 × g, 24 ºC). Each extraction solvent was collected and pooled in a PP tube (50 mL) after each cycle. After six cycles, the pooled extracts were centrifuged (1 h, 3700 × g, 24 ºC) prior to SPE. Prior to loading, SPE cartridges were conditioned with methanol (4 mL), followed by Nanopure™ water (4 mL).Pooled extraction solvent was loaded (1 mL/ min) onto Waters™ C18 Sept Pac (6 cc, 500 mg) SPE cartridges on a vacuum manifold using high-volume PP lines. After loading the samples, PP bottles, lines, and SPE cartridges were washed with 20 mM ammonium acetate (pH 3.8, 6 mL) to collect any remaining analytes. Cartridges were then dried under vacuum (1 h). Samples were eluted with two, 3 mL volumes of methanol into clean 15-mL PP tubes. Graphitized carbon black (GCB) (100 mg) was added into the elution solvent to further improve matrix removal, and then samples were vortexed and centrifuged as described above. Clean extracts were collected into a clean 15-mL PP tube; the tube with the GCB was quantitatively transferred with an additional aliquot of methanol (1 mL) and pooled with the clean extract. Extracts were evaporated to dryness under N_2 then resuspended in 250 µL of 95:5 (v/v) solution of 5 mM ammonium acetate (pH 3.8):acetonitrile. Each extract was fortified with 25 µL of internal standard solution (1 µg/ mL), MPFOA. Finally, samples were transferred to PP centrifuge tubes (1.7 mL), centrifuged (20,800 g, 15 min), transferred to inserts (PP, 300 µL), and analyzed with LC-HRMS.

Section S5: Formulas

Total Aqueous Phase removal (%) = Adsorption and Microbial removal= 100 × (C_0 − C_t, live) / C_0;

Adsorption (%) = 100 × (C_0 − C_t, abiotic control) / C_0;

Microbial removal (%) = Total Aqueous Phase removal − Adsorption.

2. Supporting Table

Table S1: Details of 5 PFAS. PFAS analytes, abbreviations, and the associated isotopically labelled surrogate used for normalization and quantification. All mass labelled standards used in this study were purchased from *Wellington Laboratories*.

**Analyte (Abbreviation)**	**Molecular formula**	**Cas No**	**Purity**	**Molecular weight**	**LOD (ppb)**	**LOQ (ppb)**	**Boiling point °C**	**Isotopically labelled (Mass labelled) surrogate**	**Structure**
potassium perfluorooctane sulfonate (PFOS)	$CF_3(CF_2)_7SO3K$	2795-39-3	>= 98%	538.22	0.62	2.06	249	$^{13}C_8$-PFOS	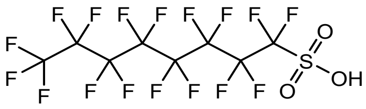
Perflouro octanoic Acid (PFOA)	$CF_3(CF_2)_6COOH$	335-67-1		414.07	0.44	1.47	192	$^{13}C_8$-PFOA	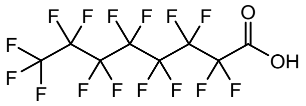
Perfluorobutane sulfonic acid (PFBS)	$CF_3(CF_2)3SO3H$	375-73-5	97%	300.10	1.99	6.64	210-212	$^{13}C_3$-PFBS	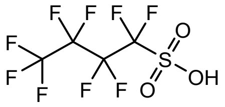
1H, 1H, 2H, 2H-perfluorooctane sulfonic acid (6:2 FTS)	$C_8H_5F_{13}O_3S$	27619-97-2	97%	428.17	0.71	2.37		$^{13}C_2$-6:2 FTS	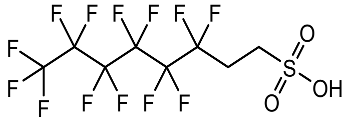
2,2,3,3-Tetrafluoro-2-(1,1,2,2,3,3,3-heptafluoropropoxy) propanoic acid HFPO dimer, acid fluoride (GenX)	$C_6F_{12}O_2$	2062-98-8		332.04	1.52	5.05	54-56	$^{13}C_3$-HFPO-DA	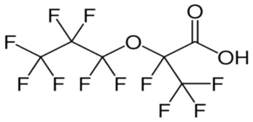

Table S2. List of 27 PFAS with their abbreviations and chemical structure screened for transformation products. LOD and LOQ provided for quantified PFAS.

Name	Abbreviation	Structure	LOD (ppb)	LOQ (ppb)
**Carboxylates**				
Perfluorobutanoic acid	PFBA	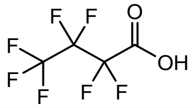	1.52	5.05
Perfluoropentanoic acid	PFPeA	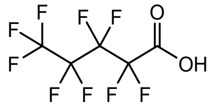	0.51	1.70
Perfluorohexanoic acid	PFHxA	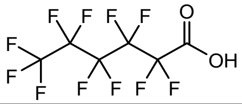	0.27	0.91
Perfluoroheptanoic acid	PFHpA	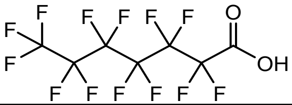	0.4	1.33
Perfluorooctanoic acid	PFOA	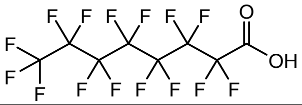		
Perfluorononanoic acid	PFNA	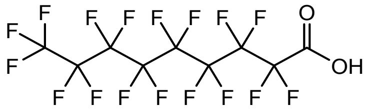		
Perfluorodecanoic acid	PFDA	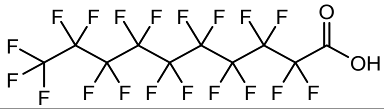		
Perfluoroundecanoic acid	PFUnA	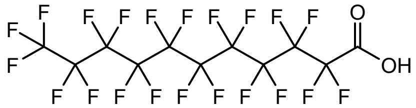		
Perfluorododecanoic acid	PFDoA	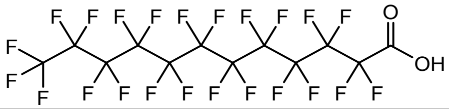		
Perfluorotridecanoic acid	PFTrDA	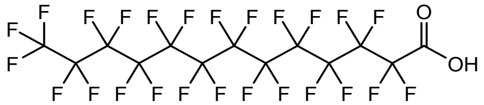		
Perfluorotetradecanoic acid	PFTeDA	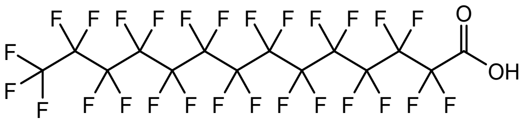		
**Sulfonates**				
Perfluorobutanesulfonic acid	PFBS	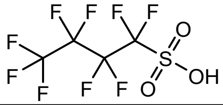	1.99	6.64
P erfluoro-1-pentanesulfonate	L-PFPeS	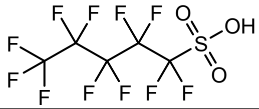	2.18	7.26

Perfluorohexanesulfonic acid	PFHxS	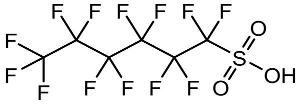	1.21	4.02
Perfluoro-1-heptanesulfonic acid	PFHpS	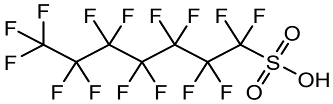	2.81	9.37
Perfluorooctanesulfonic acid	PFOS	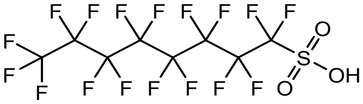		
Perfluoro-1-decanesulfonic acid	PFDS	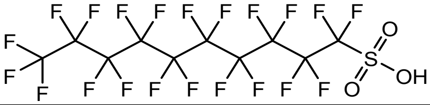		
**Sulfanamides**				
Perfluoro-1-octanesulfonamide	FOSA	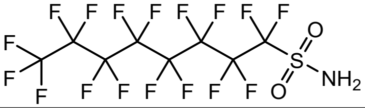		
N-methyl perfluorooctanesulfonamidoacetic acid	NMeFOSAA	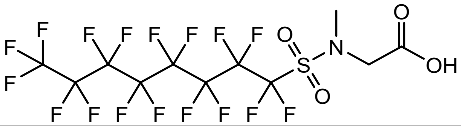		
N-ethyl perfluorooctanesulfonamidoacetic acid	NEtFOSAA	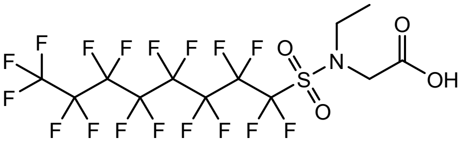		
**Fluorotelomer sulfonates**				
1H, 1H, 2H, 2H-Perfluorohexane sulfonic acid	4:2FTS	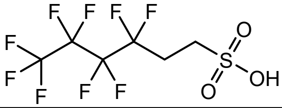		
1H, 1H, 2H, 2H-perfluorooctane sulfonic acid	6:2FTS	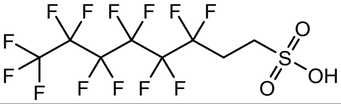		
1H, 1H, 2H, 2H-Perfluorodecane sulfonic acid	8:2FTS	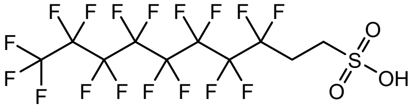		
**Ethers**				
Perfluoro-3-methoxypropanoic acid	PFMPA	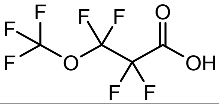		
Perfluoro-4-methoxybutanoic acid	PFMBA	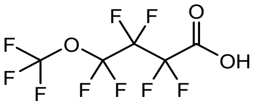		
Nonafluoro-3,6-dioxaheptanoic acid	NFDHA	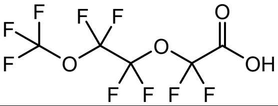		
Perfluoro(2-ethoxyethane)sulfonic acid	PFEESA	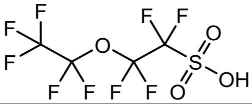		

Table S3. Anaerobic Culture Design and Operational Conditions

Sample Type	Electron Donor	Inoculum	Electron Acceptor	Number of Replicates
Experimental	Lactate	KB-1® enrichment culture	PFOA	2
			PFOS	
			6:2 FTS	
			PFBS	
			GenX	
		WWTP sludge	PFOA	
			PFOS	
			6:2 FTS	
			PFBS	
			GenX	
Abiotic Control	Lactate	Autoclaved KB-1® enrichment culture	PFOA	2
			PFOS	
			6:2 FTS	
			PFBS	
			GenX	
		Autoclaved WWTP sludge	PFOA	
			PFOS	
			6:2 FTS	
			PFBS	
			GenX	
Biotic Control	Lactate	KB-1® enrichment culture	TCE	2
		Autoclaved KB-1® enrichment culture	TCE	2

Table S4. Aerobic Culture Design and Operational Conditions

Sample Type	Inoculum	PFAS	Number of Replicates
Experimental	Mixed sludge	PFOA	3
		PFOS	
		6:2 FTS	
		PFBS	
		GenX	
	Activated Sludge	PFOA	
		PFOS	
		6:2 FTS	
		PFBS	
		GenX	
	Nitrification Sludge	PFOA	
		PFOS	
		6:2 FTS	
		PFBS	
		GenX	
Abiotic Control	Mixed sludge	PFOA	3
		PFOS	
		6:2 FTS	
		PFBS	
		GenX	

Table S5. Significant difference between biological replicates with different PFAS exposure (PFOA, PFOS, and 6:2 FTS) using different sludge type using AMOVA

Sludge Type	Day	PFAS 1	PFAS 2	p value
Mixed	0	PFOS	PFOA	0.342
		PFOS	62FTS	0.340
		PFOA	62FTS	0.332
	6	PFOS	PFOA	0.351
		PFOS	62FTS	0.347
		PFOA	62FTS	0.340
	12	PFOS	PFOA	0.335
		PFOS	62FTS	0.336
		PFOA	62FTS	0.363
	21	PFOS	PFOA	0.336
		PFOS	62FTS	0.329
		PFOA	62FTS	0.647
Activated	0	PFOS	PFOA	0.342
		PFOS	62FTS	0.665
		PFOA	62FTS	0.660
	6	PFOS	PFOA	0.669
		PFOS	62FTS	0.347
		PFOA	62FTS	0.340
	12	PFOS	PFOA	0.335
		PFOS	62FTS	0.678
		PFOA	62FTS	0.363
	21	PFOS	PFOA	0.336
		PFOS	62FTS	0.684
		PFOA	62FTS	0.303
Nitrification	0	PFOS	PFOA	0.684
		PFOS	62FTS	0.665
		PFOA	62FTS	0.660
	6	PFOS	PFOA	0.351
		PFOS	62FTS	0.347
		PFOA	62FTS	0.340
	12	PFOS	PFOA	0.335
		PFOS	62FTS	0.678
		PFOA	62FTS	0.692
	21	PFOS	PFOA	0.336
		PFOS	62FTS	1.000
		PFOA	62FTS	0.647

Table S6: PFAS identified in biosolid (before spiking with PFAS)

PFAS type	PFAS	MS (aqueous)	NS (solid)	AS (solid)
		$mg.L^{-1}$	$ng{\cdot}mg^{-1}$	$ng{\cdot}mg^{-1}$
Target short chain compound	PFHpA	0	0	0
	PFHxA	0	0	0
	PFPeA	0	0	0
	PFHpS	0	0.040	0.050
	PFHxS	0	0	0
	PFPeS	0	0.150	0.010
	PFBS	0	0.080	0.080
Parent Compound	PFOA	0	0	0
	PFOS	0	3.12	4.4
	6:2 FTS	0	0.66	0.44

Table S7: Extraction Recovery (%) by spiking biosolid samples with 1 mg/L MPFAC, 19 ES standard mix

Mass labelled Compound	Extraction Recovery (%)
PFBA	14
PFPeA	80
PFHxA	95
PFHpA	87
PFOA	80
PFBS	90
PFHxS	84
PFOS	65
4:2FTS	102
6:2FTS	85
8:2FTS	69

Table S8: Fluoride Mass Balance for PFOA at live nitrification sludge and killed control sludge at day 21

	Live Sludge		Control Sludge	
	Fluoride (mg)	% wrt initial concentration	Fluoride (mg)	% wrt initial concentration
PFOA(aq)	0.584	16.987	2.538	92.306
PFOA(s)	1.209	35.149	0.232	8.425
Short product(aq)	0.004	0.110	0.005	0.189
Short product(s)	0.018	0.535	0.000	0.000
Fluoride (aq)	0.018	0.519	0.014	0.515
Sum	1.834	53.300	2.789	101.435
Missing	1.606	46.700	0.651	-1.435

Table S9: Fluoride Mass Balance for PFOS at live nitrification sludge and killed control sludge at day 21:

	Live sludge		Control sludge	
	Fluoride (mg)	% wrt initial concentration	Fluoride (mg)	% wrt initial concentration
PFOS(aq)	0.355	13.752	1.285	49.756
PFOS(s)	1.480	57.292	1.238	47.940
Short product(aq)	0.004	0.162	0.035	1.355
Short product(s)	0.181	7.021	0.102	3.966
Fluoride (aq)	0.021	0.794	0.013	0.492
Sum	2.041	79.020	2.674	103.508
Missing	1.189	20.980	0.556	-3.508

Table S10: Rate constant k (d^{-1}) and halftime $t_{1/2}$ (d) using $\ln(C/C_0)$ vs. time (t > 6 d; C corrected by the abiotic control) with a pseudo–first-order kinetics analysis after acclimation (~6 d) for PFOA, PFOS, and 6:2 FTS across MS/AS/NS

	**PFOA**			**PFOS**			**6:2 FTS**		
	**MS**	**AS**	**NS**	**MS**	**AS**	**NS**	**MS**	**AS**	**NS**
rate constant, k (per day)	0.1185	0.0876	0.0998	0.0305	0.2663	0.2744	0.0795	0.1071	0.1089
	0.1330	0.1155	0.1076	0.0890	0.2483	0.3002	0.0817	0.1154	0.1094
	0.1346	0.0859	0.1058	0.1215	0.2278	0.2758		0.1060	0.0939
halftime (t1/2), [illegible]	5.8490	7.9110	6.9478	22.7158	2.6033	2.5257	8.7165	6.4741	6.3650
	5.2124	5.9993	6.4393	7.7868	2.7916	2.3091	8.4859	6.0070	6.3365

	5.1506	8.0705	6.5498	5.7029	3.0428	2.5128	#DIV/0!	6.5363	7.3808

Table S11: Summary of determining rate constant k (d^{-1}) using $\ln(C/C_0)$ vs. time (t > 6 d; C corrected by the abiotic control) with a pseudo–first-order kinetics analysis after acclimation (~6 d) for PFOA, PFOS and 6:2 FTS across MS/AS/NS

	Summary stats						
	**Treatment**	**n**	**Mean_k**	**SD**	**SE**	**95%CI_lower**	**95%CI_upper**
	MS	3	0.1287	0.0089	0.0051	0.1067	0.1507
	AS	3	0.0963	0.0166	0.0096	0.0550	0.1377
PFOA	NS	3	0.1044	0.0041	0.0024	0.0942	0.1147
	MS	3	0.0804	0.0461	0.0266	-0.0342	0.1949
	AS	3	0.2474	0.0192	0.0111	0.1997	0.2952
PFOS	NS	3	0.2835	0.0145	0.0084	0.2475	0.3194
	MS	2	0.0806	0.0015	0.0011	0.0669	0.0943
	AS	3	0.1095	0.0051	0.0030	0.0968	0.1222
6:2 FTS	NS	3	0.1041	0.0088	0.0051	0.0822	0.1259

Table S12: Determination of the statistical significance of rate constants across sludge types for PFOA, PFOS, and 6:2 FTS analysis of variance (ANOVA) at 95% confidence level (α= 0.05)

	**ANOVA, α=0.05**	
	**Statistic**	**p-value**
PFOA	6.8497	0.0283
PFOS	39.0498	0.0004
6:2 FTS	12.7866	0.0108

Table S13: Post hoc analysis using Tukey's HSD test (α= 0.05) to evaluate pairwise differences among the rate constants of AS-MS, MS-NS and NS-AS for PFOA, PFOS and 6:2 FTS

Tukey HSD Test, α=0.05							
	**group 1**	**group 2**	**meandiff**	**p-adj**	**lower**	**upper**	**reject**
PFOA	AS	MS	0.0323	0.0278	0.0044	0.0602	TRUE
	AS	NS	0.0081	0.6676	-0.0198	0.0360	FALSE
	MS	NS	-0.0243	0.0824	-0.0522	0.0036	FALSE
PFOS	AS	MS	-0.1671	0.0012	-0.2424	-0.0918	TRUE
	AS	NS	0.0360	0.3683	-0.0392	0.1113	FALSE
	MS	NS	0.2031	0.0004	0.1279	0.2784	TRUE
6:2 FTS	AS	MS	-0.0289	0.0104	-0.0481	-0.0097	TRUE
	AS	NS	-0.0054	0.5933	-0.0226	0.0118	FALSE
	MS	NS	0.0235	0.0241	0.0042	0.0427	TRUE

3. Supporting Figures

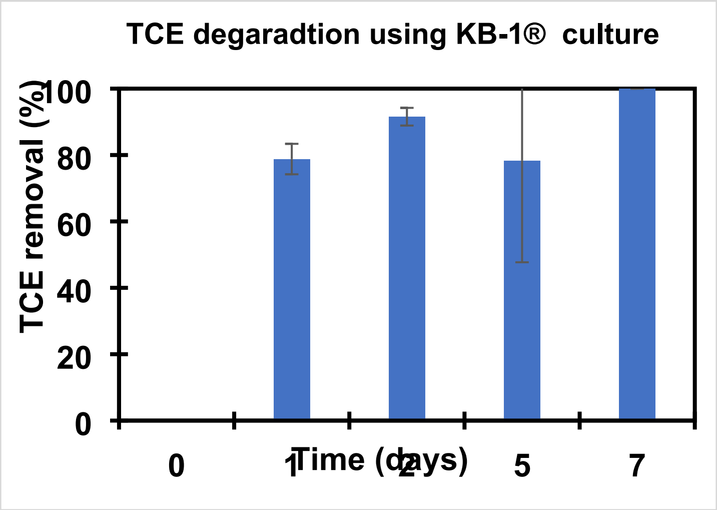

Figure S1. TCE degradation over 7 days using commercially available KB-1® enrichment culture. Removal is calculated with respect to control. This study was done as a proof of biotic activity of KB-1® enrichment culture.

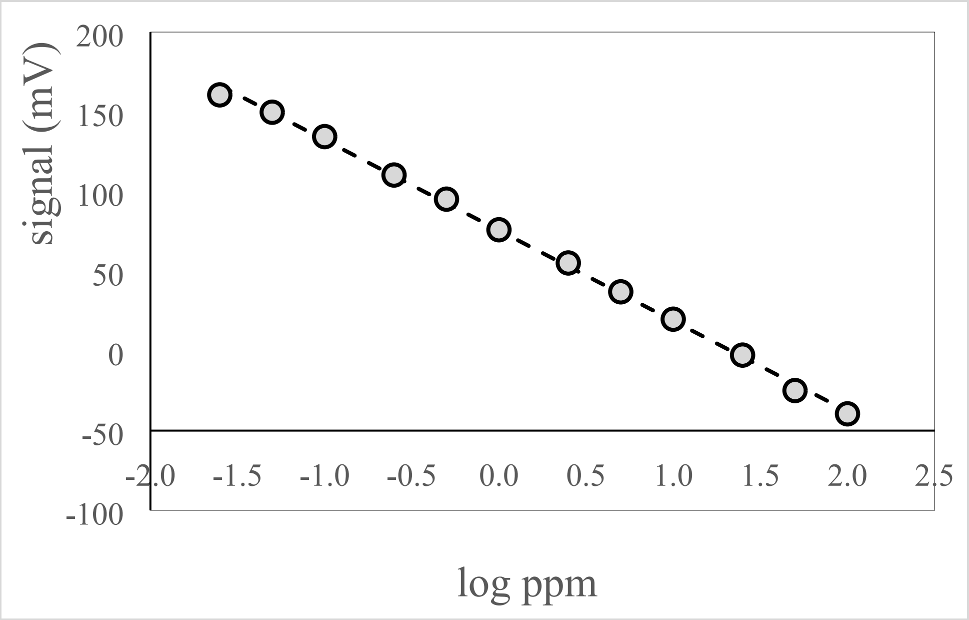

Figure S2. Calibration curve of fluoride (F^-) using ion-selective electrode (ISE) for limit of detection (LOD) calculations.

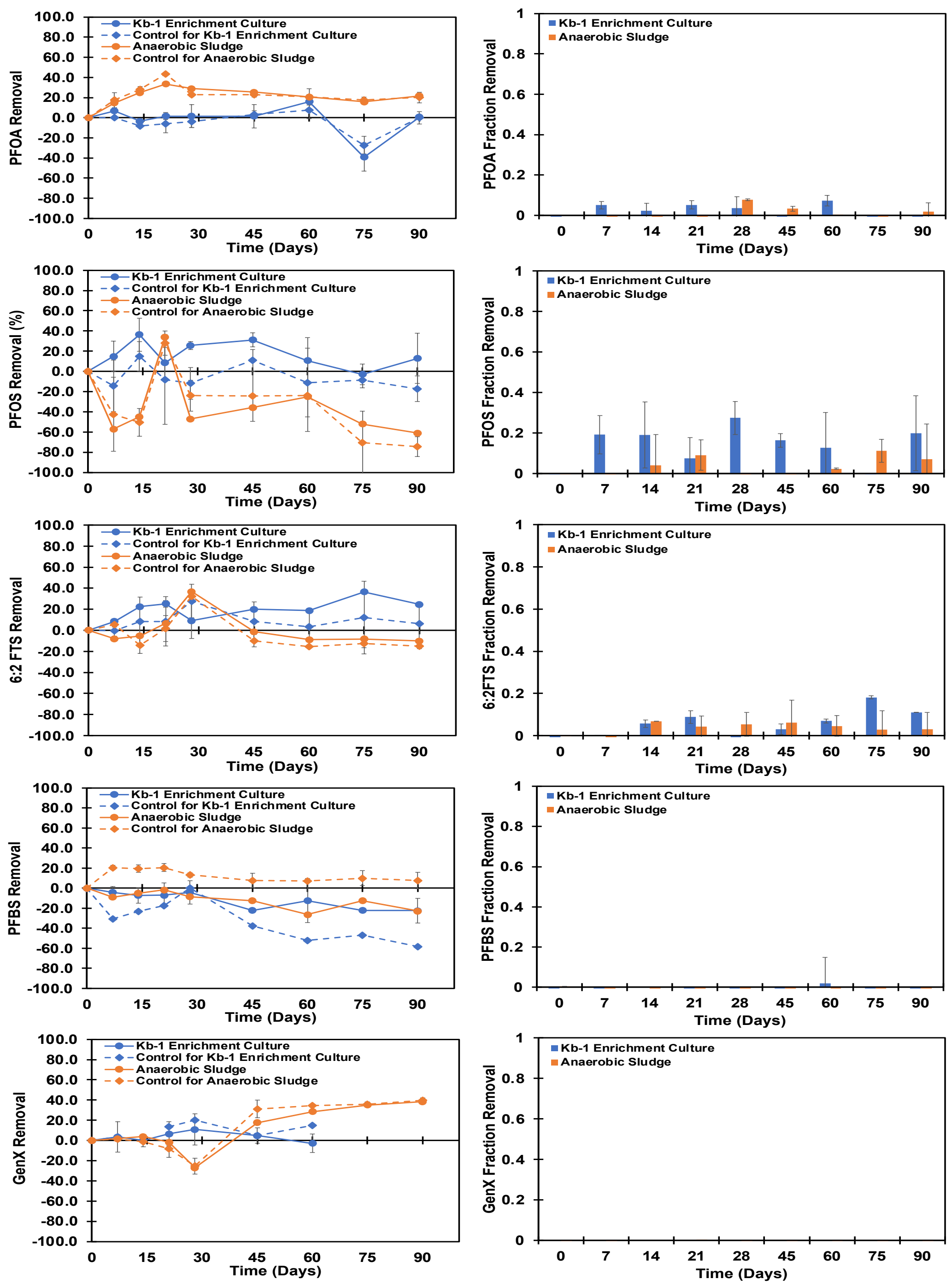

Figure S3. Anaerobic Degradation of PFOA, PFOS, 6:2 FTS, PFBS and GenX with KB-1® enrichment culture (blue) and anaerobic sludge (orange) over 90 days where the circle indicates biotic samples and diamond indicates heat inactivated controls.

Left graphs show removal of parent compound in comparison with initial dosage of PFAS where removal was calculated as **% Removal= (1-** $\frac{Concentration\ at\ time\ t = t}{concentration\ at\ time\ t = 0}$**)** $\times$ **100**. Right graphs represent fraction removal of PFAS with respect to heat inactivated control to account for any

abiotic activity where removal was calculated as **Fraction Removal= (1-** $\frac{Concentration\ of\ sample\ at\ time\ t=t}{concentration\ of\ control\ at\ time\ t=t}$)

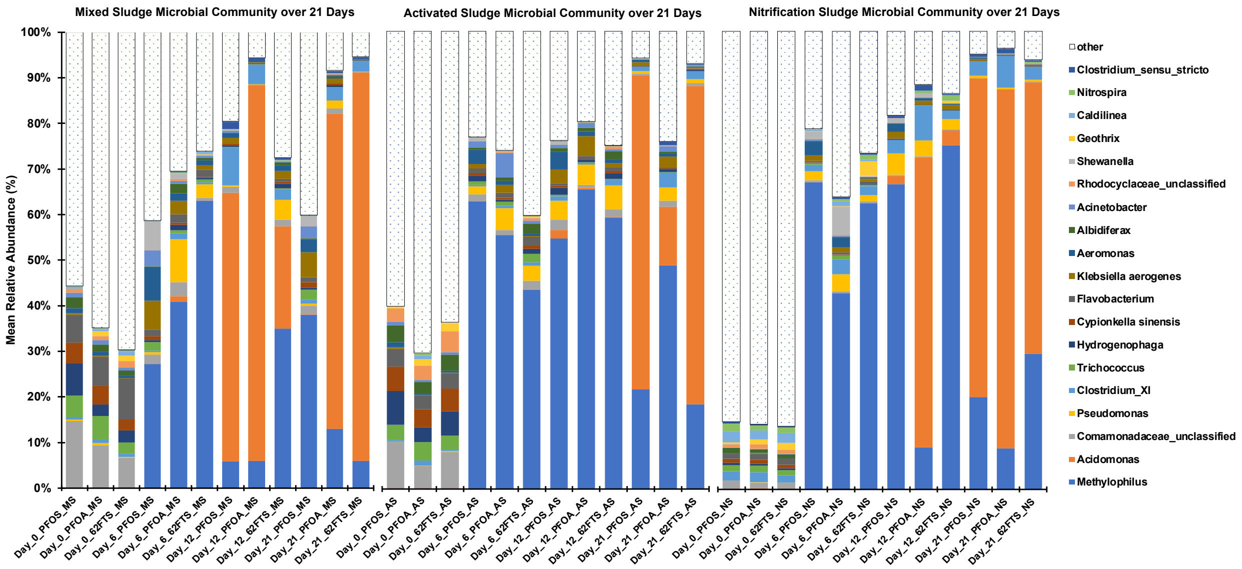

Figure S4. Mean relative abundance for the top 1% OTUs for mixed and activated sludge and 0.5% OTUs for nitrification sludge totaling 19 OTUs are presented over 21 days of incubation. Each bar represents the average microbial community observed under two replicates of each of PFOA, PFOS and 6:2FTS with each of mixed, activated and nitrification sludge incubation at each time point. Across different sludge types, the inoculum had a higher diversity that converged to a selective handful microbial community after 21 days study period.

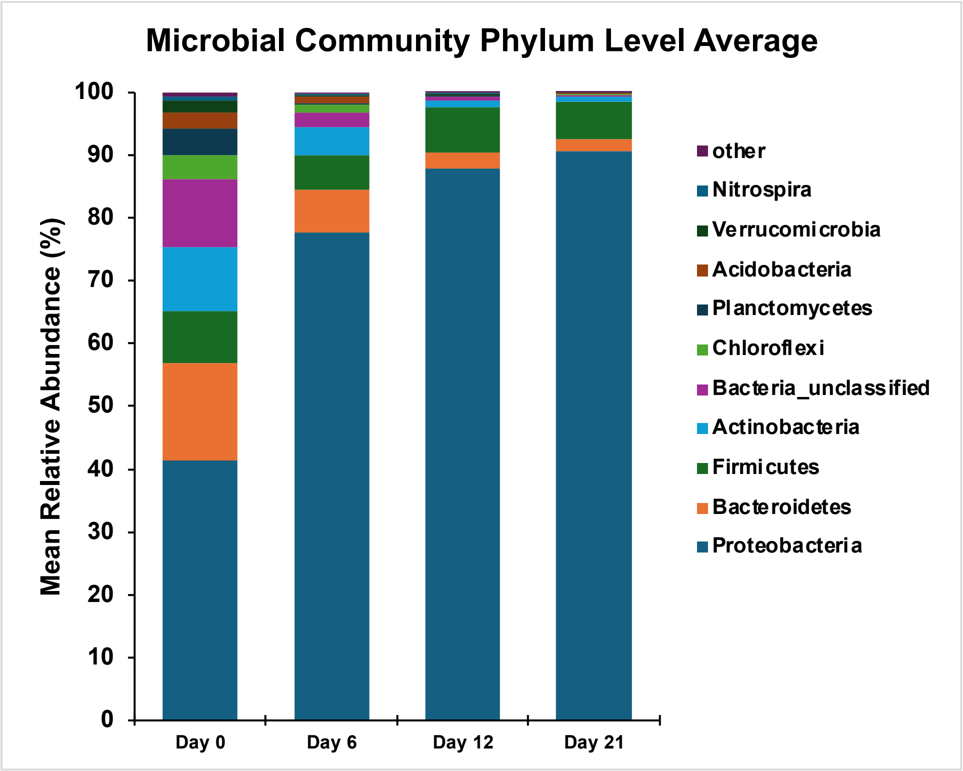

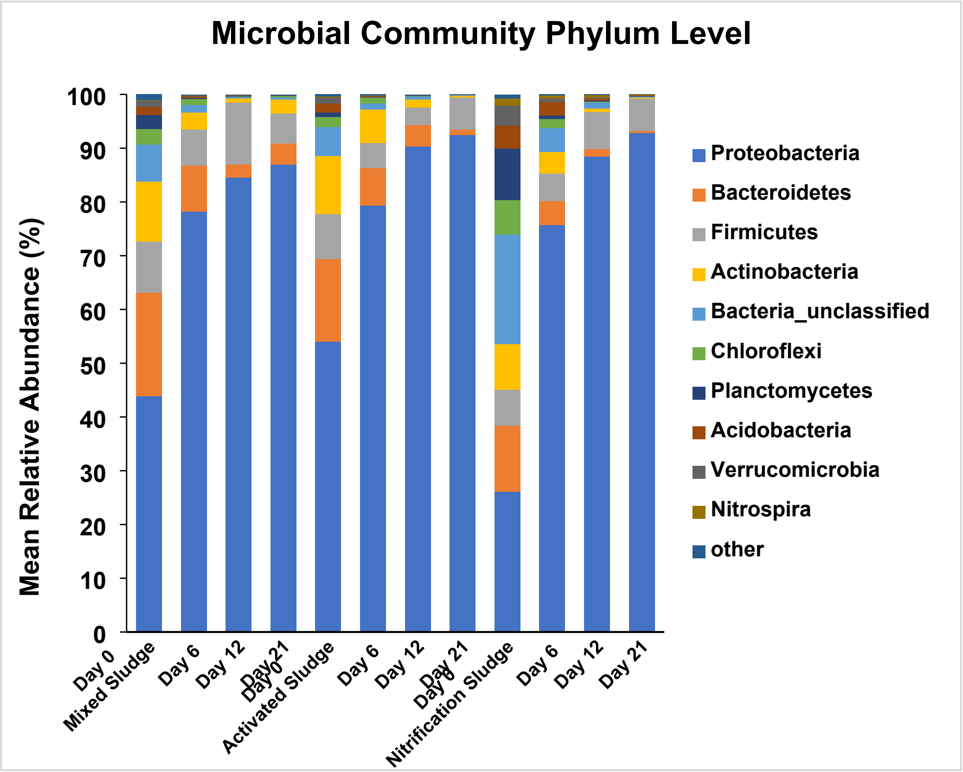

Figure S5. Mean Relative abundance of Phylum level taxa (top) averaged across all sludge type and all PFAS type at each time point over 21 days and (bottom) averaged across all PFAS type at each time point over 21 days in each sludge type.

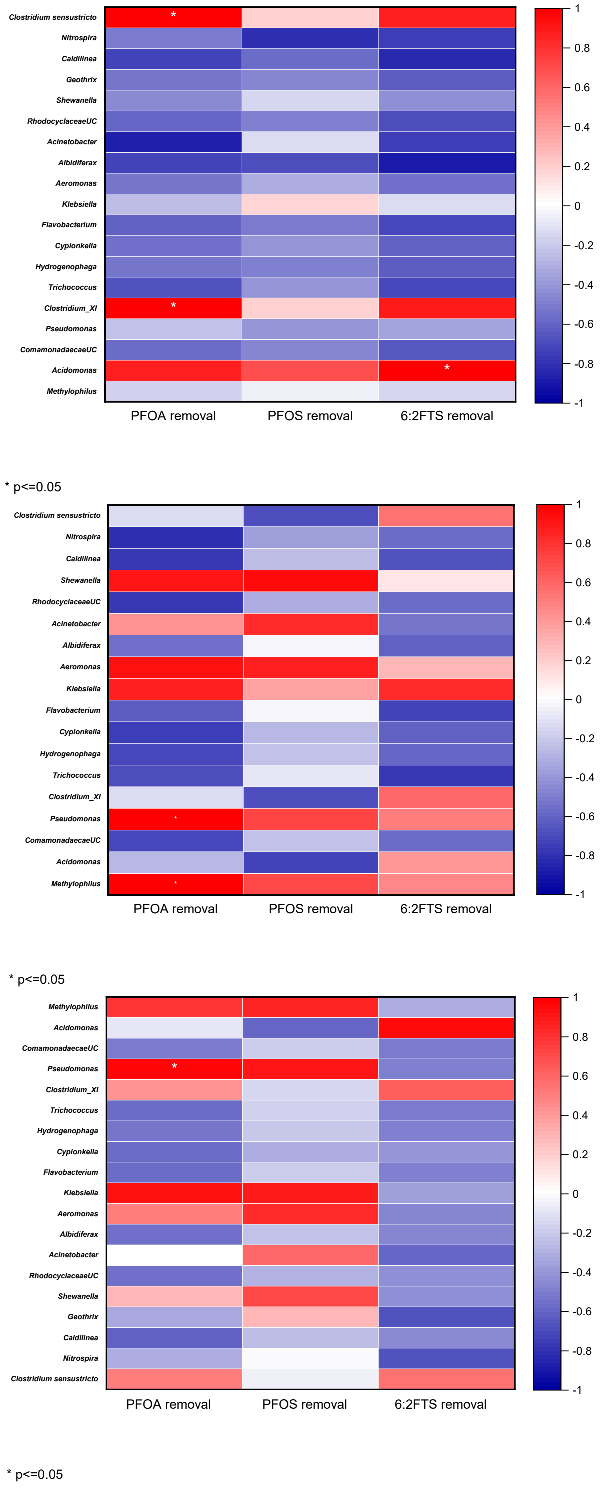

Figure S6. Pearson correlation plot between microbial community (top OTU) under (top) mixed sludge, (middle) activated sludge, and (bottom) nitrification sludge with PFAS removal over 21 days.

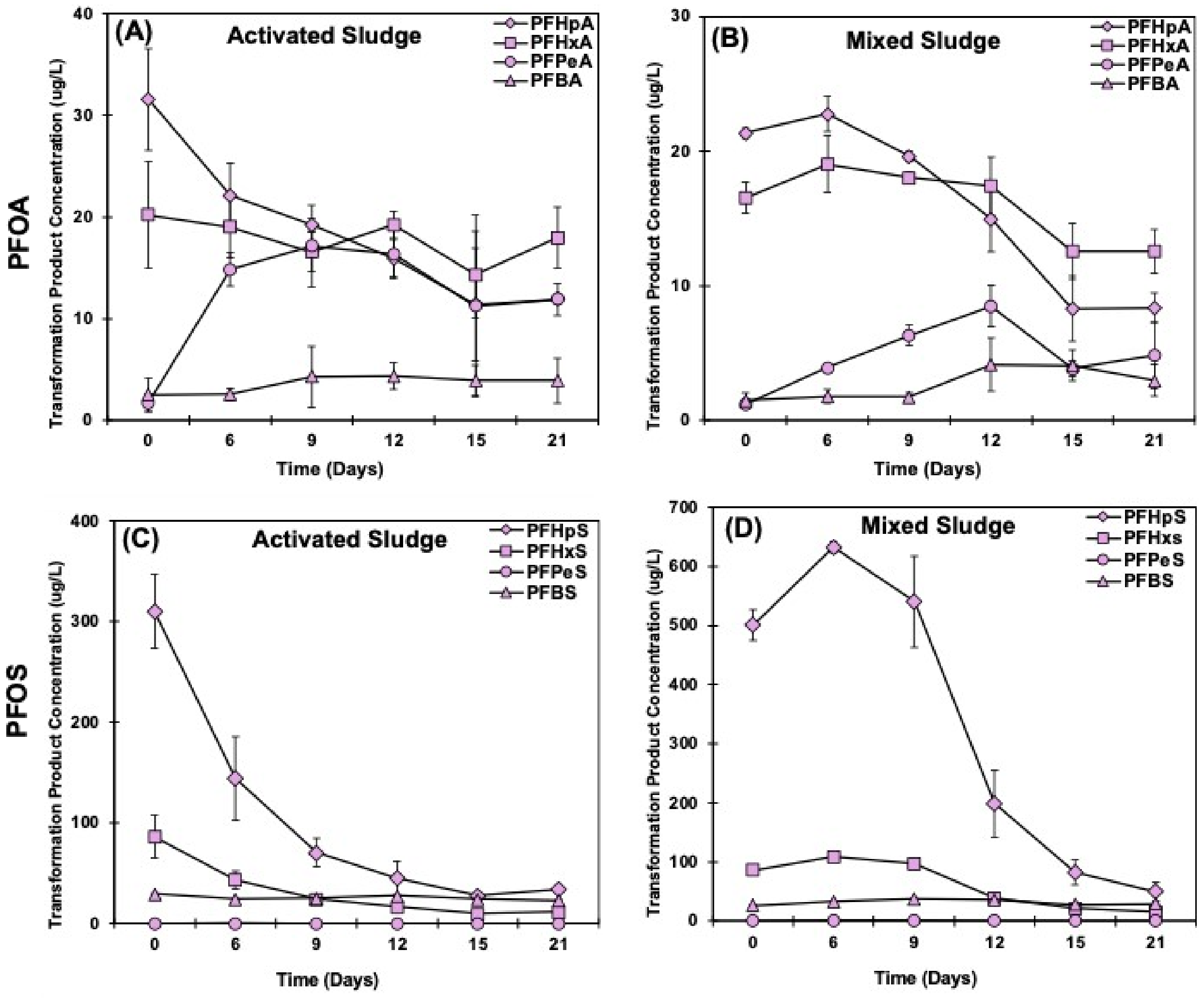

Figure S7. Transformation of PFOA under A) activated sludge, B)mixed sludge and PFOS under C) activated sludge, D)mixed sludge into shorter chain (C7-C4) PFAS in aqueous phase over 21days of aerobic incubation. Significant generation of PFPeA and PFBA is observed from PFOA degradation over 21 days.

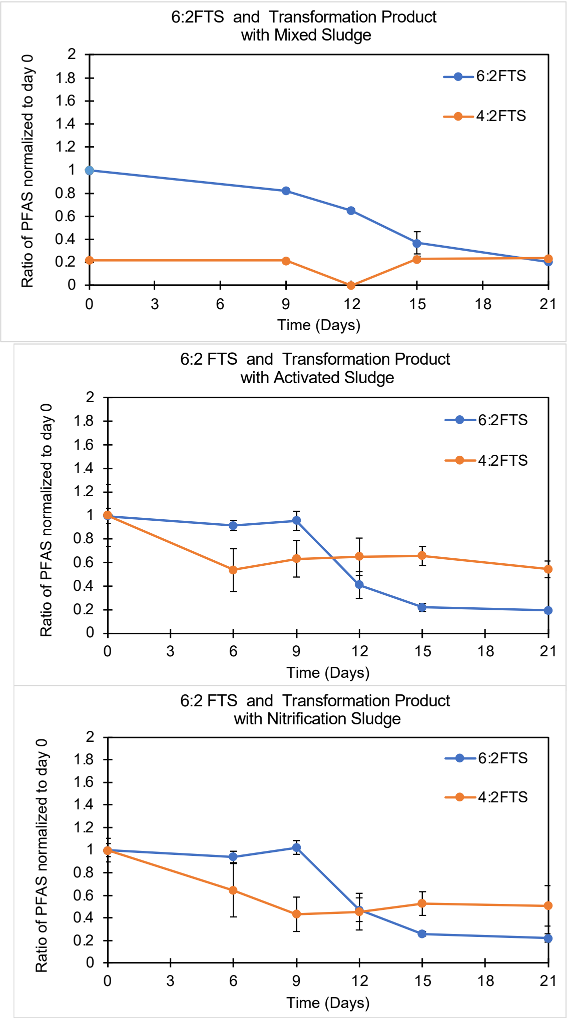

Figure S8. Ratio of 6:2 FTS and transformed product normalized to day 0 under incubation with mixed sludge, activate sludge and nitrification sludge. Only 4:2 FTS was observed but that too was always present below than its initial concentration.

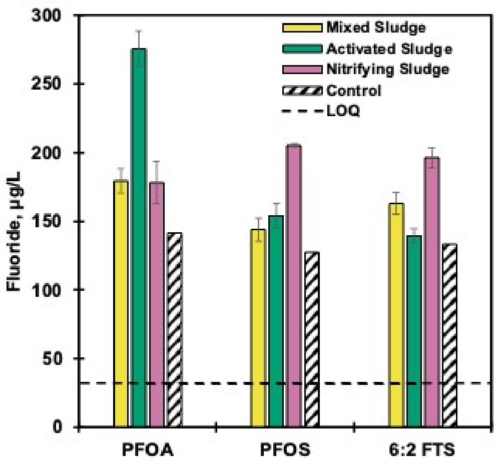

Figure S9. Detection of Fluoride in aqueous phase at day 21 (endpoint) in mixed sludge, activated sludge, nitrification sludge and autoclaved control for PFOA, PFOS and 6:2FTS, respectively. Fluoride ion concentrations were measured using a fluoride ion selective electrode (ISE). Limit of detection (LOD) and limit of quantitation (LOQ) of the ISE was 28.4 and 31.8 $\mu g \cdot L^{-1}$, respectively.

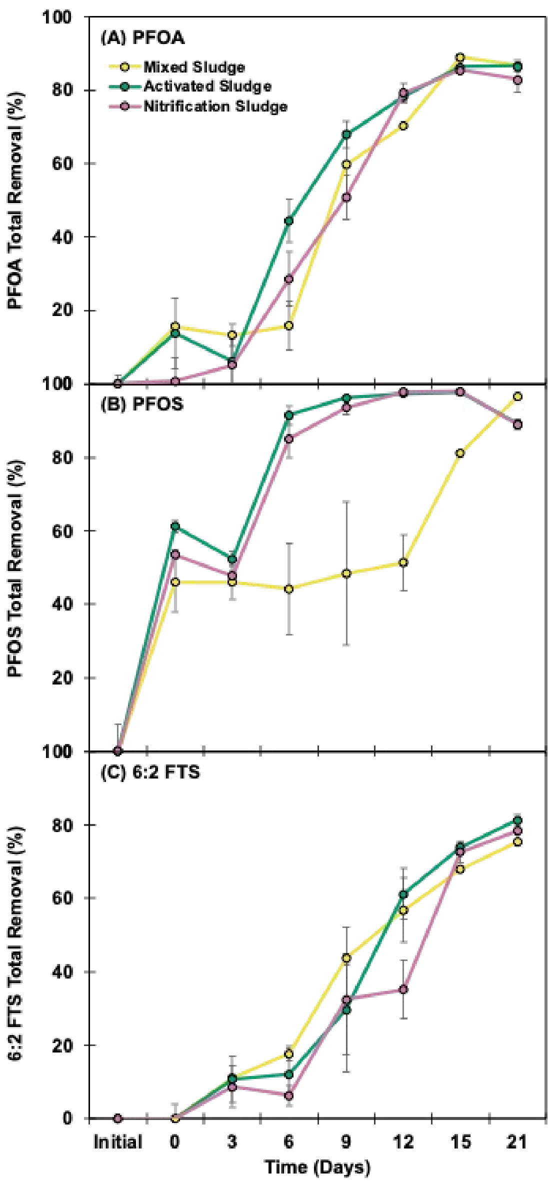

Figure S10 : PFOA,PFOS and 6:2 FTS total removal from aqueous phase with respect to initial concentration over 21-day incubation period across all sludge conditions. The total removal is a combination of adsorption, biotic accumulation, and transformation.

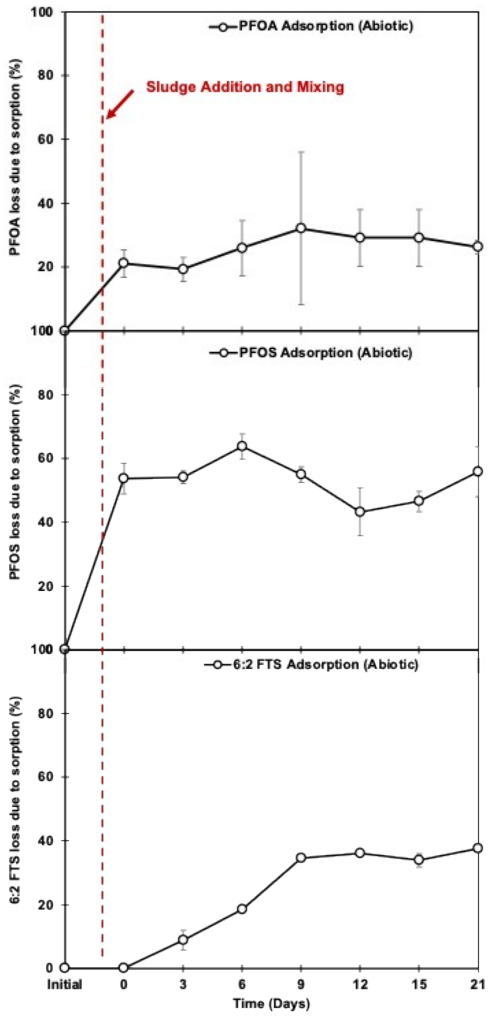

Figure S11: PFOA, PFOS, and 6:2 FTS loss (in %) from initial concentration over 21-day incubation period in abiotic control due to adsorption. For PFOA and PFOS, maximum removal (=loss) occurred right after mixing with sludge, and for 6:2 FTS, the removal reached equilibrium slowly over 9 days.

Reference:

Brotsch, J. (2017) Trichloroethylene (TCE) Sorption to Organic Matter in Sedimentary Rocks of the Newark Basin, University at Buffalo, ProQuest Dissertations Publishing.

Dickman, R. A., & Aga, D. S. (2022). Efficient workflow for suspect screening analysis to characterize novel and legacy per- and polyfluoroalkyl substances (PFAS) in biosolids. *Analytical and Bioanalytical Chemistry*, *414*(15), 4497–4507. https://doi.org/10.1007/s00216-022-04088-2
